# Reelin Alterations, Behavioral Phenotypes, and Brain Anomalies in Schizophrenia: A Systematic Review of Insights From Rodent Models

**DOI:** 10.3389/fnana.2022.844737

**Published:** 2022-03-24

**Authors:** Ana C. Sánchez-Hidalgo, Celia Martín-Cuevas, Benedicto Crespo-Facorro, Nathalia Garrido-Torres

**Affiliations:** ^1^Spanish Network for Research in Mental Health (CIBERSAM), Madrid, Spain; ^2^Seville Biomedical Research Centre (IBiS), Seville, Spain; ^3^Department of Psychiatry, School of Medicine, University Hospital Virgen del Rocío-IBiS, Seville, Spain

**Keywords:** animal models, reelin, brain, neurodevelopment, extracellular matrix

## Abstract

Reelin is an extracellular matrix glycoprotein reduced in brain regions (the prefrontal cortex and the hippocampus) of patients with schizophrenia. There are diverse rodent models of schizophrenia that mimic patient symptoms based on various causal theories; however, likely shared reelin alterations have not yet been systematically assessed in those models. A systematic review of the literature was conducted following the Preferred Reporting Items for Systematic Reviews and Meta-Analyses (PRISMA) model. Articles focused on psychotic disorders or schizophrenia and their relationship with reelin in rodent models were selected. Data (first author, publication year, results, both open field and prepulse inhibition test results, and type of reelin alteration) were extracted in duplicate by two independent reviewers. The 37 reviewed articles reported about various schizophrenia models and their reelin alterations, brain morphology, and behavioral defects. We conclude that reelin is an altered preclinical biomarker common to all models included, mainly prenatal or genetic models, and a key protein in schizophrenia disease, making the reelin signaling pathway in prenatal stages a target of special interest for future preclinical and clinical studies. All models presented at least one of the four described reelin alteration types.

**Systematic Review Registration:** [https://www.crd.york.ac.uk/prospero/display_record.php?ID=CRD42021210568], identifier [CRD42021210568].

## Highlights

- RELN alterations seem to be a common biomarker contributing to the face validity of schizophrenia rodent models.
- RELN signaling pathway in prenatal stages turns to be crucial for preclinical and clinical studies in schizophrenia.
- The variability found in behavioral tests, highlights the need to establish behavioral protocols to compare consistent results.
- Most of reelin alterations were described in the prefrontal cortex and hippocampus, both regions highly involved in schizophrenia disease.
- Sexual dimorphism of schizophrenia is a relevant aspect and future studies should include female and male rodents.

## Introduction

Reelin (RELN) is an extracellular matrix glycoprotein that is essential for neuronal migration and laminar positioning during brain development that regulates dendritic growth and spine formation, synaptogenesis and synaptic plasticity (Teixeira et al., 2011; Wasser and Herz, 2017; Yamakage et al., 2019; Jossin, 2020), and adult neurogenesis (Bosch et al., 2016). RELN is mainly produced in Cajal-Retzius neurons during development and in interneurons in the cerebral cortex and the hippocampus during postnatal stages (Negrón-Oyarzo et al., 2016; Wasser and Herz, 2017; Yamakage et al., 2019; Jossin, 2020; Tsuneura et al., 2021). RELN is a ∼400-kD protein coded by a 450 kbp gene located in human chromosome 7q22 and murine chromosome 5 that binds to Apolipoprotein E receptor 2 (ApoER2) and very-low-density lipoprotein receptor (VLDLR) (Bosch et al., 2016; Wasser and Herz, 2017), inducing phosphorylation of disabled homolog-1 (Dab1), which modulates a downstream cascade (Sakai et al., 2016; Tsuneura et al., 2021). RELN proteolytic cleavage is mediated by a disintegrin and metalloproteinase with thrombospondin motifs (ADAMTS) metalloprotease (Yamakage et al., 2019). It has been identified in a spontaneous autosomal recessive mutant mouse strain reeler [heterozygote reeler mouse (HRM)] (Falconer, 1951).

In human, the alteration of the normal development of these *RELN*-regulated brain processes and functions has been associated with neurodevelopmental disorders such as autism, intellectual disability, and schizophrenia (SCZ) (Jossin, 2020). Many of the candidate genes in SCZ are involved in neurodevelopment and neuroplasticity processes and thus are crucial during brain development, supporting the notion that SCZ is a neurodevelopmental disorder (Howell and Pillai, 2016; Cleynen et al., 2020; Hannon et al., 2021). It has been described as an important link between prenatal stress and cellular and physiological alterations observed in SCZ (Negrón-Oyarzo et al., 2016). Previous studies have strongly shown RELN genetic anomalies and signaling impairments associated with SCZ (Impagnatiello et al., 1998; Fatemi et al., 2005; Imai et al., 2017; Arioka et al., 2018; Marzan et al., 2021). In addition, postmortem studies have also revealed that the expression of RELN is reduced at cortical and subcortical regions of patients with SCZ (Guidotti et al., 2000; Eastwood and Harrison, 2006; Nullmeier et al., 2011; Howell and Pillai, 2016). All this evidence seems to point to the relationship between alterations in RELN and the risk of SCZ (Folsom and Fatemi, 2013).

Schizophrenia rodent models are clearly very valuable preclinical tools to investigate the neurobiological basis of the disorder and provide an excellent way to infer the underlying mechanisms of SCZ in human (Radonjić et al., 2013; Steeds et al., 2015; Winship et al., 2019; Ang et al., 2021). Due to the diversity of available SCZ rodent models (over 20 types have been described), fitting into different induction categories (developmental, drug-induced, lesion, or genetic manipulation), the study of shared biological alterations that are present in many of these models can be of great value to describe pathophysiologic mechanisms or preclinical biomarkers of the disease.

**FIGURE 1:**
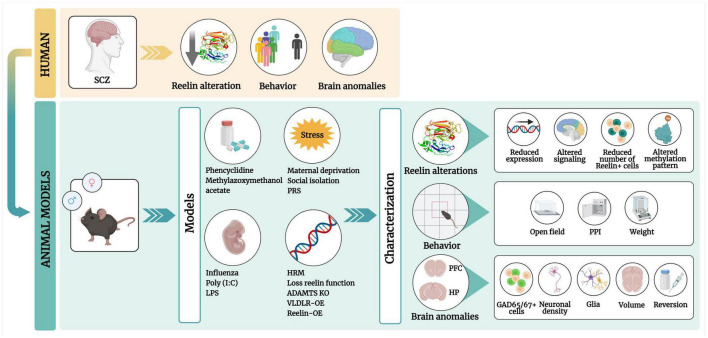
Graphical abstract of this review. Patients with schizophrenia (SCZ) have low RELN levels in the brain, with behavioral defects and brain anomalies. These phenotypes can be mimicked in pharmacological, maternal immune activation, stress, and genetic rodent models. These models include four types of RELN alterations, behavioral phenotypes [in the open field and prepulse inhibition (PPI) tests] and brain anomalies in the prefrontal cortex (PFC) and the hippocampus (HP) (regions). PCP, Phencyclidine; MAM, methylazoxymethanol; Poly(I:C), polyinosinic:polycytidylic acid; LPS, lipopolysaccharide; PRS, prenatal restraint stress; HRM, heterozygous reeler mouse; OE, overexpression. Created with Biorender.com.

We aimed to systematically review the literature on RELN anomalies that have been described in a diversity of SCZ rodent models. If RELN impairments are repeatedly observed, we may propose that these might be considered as a preclinical biomarker of the illness. We also focused on the aspects of brain morphology and behavioral effects in all models, ranging from pharmacological to stress and genetics (Figure 1).

## Method

A systematic review was conducted following the Preferred Reporting Items for Systematic Reviews and Meta-analyses (PRISMA) statement (Moher et al., 2009) and a protocol was registered in PROSPERO (CRD42021210568).

### Search Strategy

A systematic review of the literature was carried out following the PRISMA model. The terms used for the search were: “psychotic disorder” or “schizophreni*” and “reelin” and “brain” or “cerebr*” or “blood” or “mice” or “rat” or “human” or “patient” (for the precise search algorithm used in the PubMed and Web of Science databases see Supplementary Material 1). The search was conducted in September 2021. Articles included in the analysis focused on psychotic disorders or SCZ and their relationship with RELN and rodent models. The articles were evaluated by two independent reviewers. We also made a cross-reference search of included relevant studies and previous reviews and contacted study authors and experts for data clarification.

### Eligibility

The inclusion criteria were selected to systematize the search in the databases so that only articles of interest on the subject of behavioral and brain phenotypes due to RELN alteration in SCZ rodent models would be retrieved. These inclusion criteria were studies on (i) murine models of SCZ, (ii) evaluation of RELN expression in SCZ, (iii) phenotypic alterations in the brain: (structure, molecular level, proteins), (iv) behavioral disturbances observed in prenatal, early postnatal, or adulthood, (v) and comparison with a control group. The exclusion criteria were studies (i) on humans, animal models other than rodents, or cells, (ii) published before 2010 (studies were excluded because their results were already present in the selected articles [Supplementary Material 2]), (iii) on extracellular matrix (ECM) alterations other than RELN, (iv) on genetic studies and other psychiatric disorders, (v) on hormones (oxytocin, serotonin, etc.), blood, other proteins, (vi) on uncontrolled studies, and (vii) on phenotypic alterations in organs other than the brain, (viii) and on studies not published in peer review journals.

### Study Selection

Two independent reviewers (CM-C and ACS-H) screened the titles and abstracts to identify studies that met the inclusion criteria outlined above using Rayyan (Ouzzani et al., 2016) software. The same researchers then reviewed the eligible full texts. The final list of articles was agreed to by consensus. Disagreements on eligibility were resolved by discussions with two additional reviewers (BC-F and NG-T).

### Data Extraction, Synthesis, and Quality Assessment

The following information was extracted in duplicate from each study: first author, year, rodent model, species, methods, and type of RELN alteration. The open field test (distance traveled and time spent in the center) and prepulse inhibition test results (inhibition percentage) were selected for a meta-analysis because of their relevance in SCZ when possible. Whereas, a meta-analysis was not performed due to outcome heterogeneity, lack of reported original data, and variability found in behavioral tests and protocols.

Study quality was assessed using the Systematic Review Center for Laboratory Animal Experimentation (SYRCLE) risk of bias tool, which is the adapted version for animal studies of the Cochrane Risk of Bias tool (Hooijmans et al., 2014). Outcome measures were extracted separately by two of the researchers and categorized in five areas: 1. population (rat or mouse); 2. sex; 3. rodent model; 4. behavior; and 5. RELN alteration. The Supplementary Material was used for additional data and authors were contacted to request additional information.

## Results

### Data Analysis: Studies Included and Excluded

**FIGURE 2:**
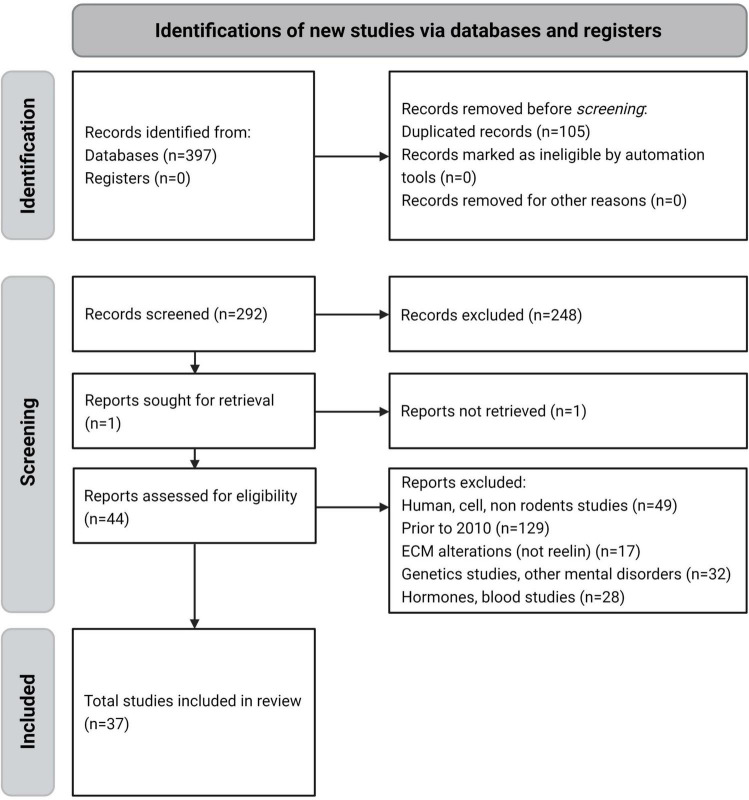
PRISMA flow diagram of the reviewing process – systematic selection for inclusion or exclusion.

A total of 397 articles were retrieved from the databases. Of these, 105 duplicate references found by cross-referencing were excluded, and 255 more were excluded for the following reasons: reported on human, cell, or non-rodent studies (49 articles), were written before 2010 (129), were on ECM alterations not related to RELN (17), were on genetic or other mental disorders (32), and were on hormone or blood studies (28). The remaining 37 studies were included in the final analysis (Figure 2).

### Risk of Bias

All studies were found to have an unclear or a low risk of bias according to the SYRCLE risk of bias tool (Supplementary Material 4). Most studies described animal and housing characteristics and reported some attrition bias. However, information on sequence generation, allocation concealment, caretaker/researcher blinding, and random outcome assessment was barely reported.

### Reelin Alteration in Rodent Models of Schizophrenia

Previous literature reporting on RELN alterations described in various SCZ rodent models, as well as their behavioral and brain phenotypes, are reported below. Only those SCZ rodent models in which RELN biology has been investigated are included in our systematic review [for the main findings of studies in this review (model type, animal, methods, analysis, results, and RELN alterations) see Supplementary Material 3].

#### Pharmacological Models

Pharmacological models evaluate the effect of administering a drug that causes SCZ-like symptoms in animals (Steeds et al., 2015). This review included two pharmacological models, phencyclidine (PCP) (Radonjić et al., 2013), and exposure to methylazoxymethanol (MAM) (Matricon et al., 2010; Table 1).

**TABLE 1:** Principal findings of pharmacological model studies included in this review.

	Animal model	Author	Specie	Sex	Age	Behavior characterization	Methods	Analysis	Results	Rescue experiments	Reelin alteration
Pharmacological models	PCP	Radonjić et al., 2013	Rat	Male	P70		Rat injected with 10 mg/kg at P2, P6, P9, and P12.	Immunohistochemical staining (NeuN, PV, calretinin, somatostatin, reelin, VGAT). Western blot (NRG-1).	Reduced neurons density in CA3 and dentate gyrus and interneuronal populations. Decreased reelin- and somatostatin- positive cells PFC and HP. PCP increased NRG-1 protein expression in cortex and HP.		↓ reelin positives cells in PFC and HP
	MAM	Matricon et al., 2010	Rat	Male and female	6-month-old		Rat exposed at E17.	Immunohistochemistry (NeuN, GFAP, and reelin). Morphometric analysis. Methylation analysis. RNA expression.	Reduced entorhinal cortex volume, HP and mediodorsal thalamus (decreased neuronal soma). Laminar disorganization and neuronal clusters in entorhinal cortex. Reduced reelin methylation in HP.		↓ reelin methylation in HP

*PCP, phencyclidine; MAM, methylazoxymethanol.*

##### Phencyclidine

The N-methyl-D-aspartate receptor (NMDAr) hypofunction has been hypothesized as a critical component of SCZ pathophysiology, promoting deficits in gamma-aminobutyric acid (GABA)ergic signaling. PCP, a dissociative anesthetic that induces SCZ-like psychosis with positive and negative symptoms and cognitive dysfunction, is a non-competitive antagonist of NMDAr (Radonjić et al., 2013; Grayson et al., 2016).

In the postnatal stages, RELN expressed by GABAergic interneurons modulates NMDAr activity. Radonjić et al. (2013) were the first to describe a significantly reduced density of RELN-positive cells in the retrosplenial cortex (82% of the control value) and CA1 and CA3 hippocampus subregions (68 and 46% of the control value, respectively) in P70 male rats. This alteration contributes to the face validity of PCP as a SCZ rodent model (Radonjić et al., 2013). The model also shows a decreased density of reelin-positive cells in the hippocampus and the cortex (Radonjić et al., 2013) and behavioral defects, mainly cognitive alterations (i.e., impaired acquisition of spatial learning in the Morris water maze, reduced rate of learning in the delayed spontaneous alternation test, and impaired ability to shift attentional set in the attentional set-shifting task) (Radonjić et al., 2013; Grayson et al., 2016).

##### Exposure to Methylazoxymethanol

Methylazoxymethanol is a neurotoxin that reduces DNA synthesis. It is used in rodent models to mimic some psychiatric diseases, such as SCZ (Matricon et al., 2010). The morphometric, cytoarchitectural, neuromorphometric, and ventricular enlargement effects of MAM embryonic day 17 (E17) treatment on the brain were evaluated.

Matricon et al. (2010) studied RELN involvement in the MAM mechanism and its neuropathological effects in 6-month-old male and female rats. The number of RELN positive cells and *reln* expression levels were the same in MAM and control animals. However, RELN methylation in the hippocampus was decreased, providing evidence of the involvement of RELN. This was determined with the amplification of 252bp sequence in the *reln* promoter that allowed the determination of the methylation level of CpG regions (Matricon et al., 2010). The brains of these rats had a significantly smaller volume and thickness. A non-significant enlargement of the lateral ventricles was also observed.

#### Maternal Immune Activation Models

Epidemiological studies have suggested that maternal immune activation (MIA) is linked to the development of SCZ (Ghiani et al., 2011; Harvey and Boksa, 2012; Nouel et al., 2012; Ratnayake et al., 2012; Giother with the Viravanoli et al., 2014; Fatemi et al., 2017; Conway and Brown, 2019; Aguilar-Valles et al., 2020). Exposure to prenatal infection in critical stages of gestation is an environmental risk factor for the development of this neuropsychiatric disorder and can lead to alterations in GABAergic and glutamatergic signaling systems as well as RELN and inflammatory mediators. Early gestation periods present a higher risk of development of behavioral impairments (Ghiani et al., 2011) usually found in patients with SCZ (Giother with the Viravanoli et al., 2014; Fatemi et al., 2017).

There are various rodent models for studying the effects of exposure to viral or bacterial agents during development (Ghiani et al., 2011; Harvey and Boksa, 2012). Three MIA models, infection with a mouse-adapted human influenza virus (A/NWS/33 [H1N1]), lipopolysaccharide (LPS), and polyinosinic-polyribocytidilic acid (Poly(I:C)) are reviewed below (Table 2).

**TABLE 2:** Principal findings of MIA model studies included in this review.

	Animal model	Author	Specie	Sex	Age	Behavior characterization	Methods	Analysis	Results	Rescue experiments	Reelin alteration
Maternal immune activation models	Influenza	Fatemi et al., 2017	Mouse	Male	P0, P14, P35, and P56		Mouse offspring. Maternal exposure to H1N1 at E16.	Western blot (reelin, GABAr, FMRP, mGluR5, GAD65/67, and Vldlr) in cerebellum.	Altered FMRP expression (increased at P0 and P14), VLDLR (decreased at P14, increased at P35) and GAD65/67 (increased at P35). Impaired FMRP, glutamatergic and reelin signaling.		Impaired reelin signaling
	Poly(I:C)	Harvey and Boksa, 2012	Mouse	Male and female	P28		Mouse offspring. Maternal injection of 20 mg/kg at GD9.	Immunohistochemistry (NeuN, reelin and GAD67). Body weight control.	Decreased number of reelin- positive cells in dorsal stratum oriens. Increased GAD67 expression in ventral stratum oriens in females. Weight gain differences.		↓ reelin positives cells in stratum oriens
		Ratnayake et al., 2012	Mouse	Male and female	P1, P100	Open field, novel object recognition, elevated plus maze, rotarod.	Mouse offspring. Maternal injection of 0,5 mg/kg at GD20.	Immunohistochemistry and histology (reelin, GFAP, and Iba1). Behavioral characterization.	Impairment of non-spatial memory, learning and motor activity. Reduced reelin-positive cells and increased GFAP expression, and increased number of activated microglia in HP.		↓ reelin positives cells in HP
		Giother with the Viravanoli et al., 2014	Mouse	Male	P70		Mouse offspring. Maternal injection of 1 mg/kg at GD9 with sub-cronic stress.	Immunohistochemistry (PV and reelin).	Decreased number of GABAergic interneurons in ventral dentate gyrus (exposure to Poly(I:C) and stress). Stress increases reelin expression in control offspring and decreases reelin expression in the rodent model (exposure to Poly(I:C) and stress).		↓ reelin positives cells in HP
	LPS	Ghiani et al., 2011	Rat	Male and female	E18		Rat offspring (E18). Maternal injection of 200ug at GD15 and GD16.	Immunohistochemistry and staining.	Increased thickness of the cortical plate. Abnormal distribution of immature neuronal markers and lower expression of progenitor markers. Reduced levels of Reelin and GLAST. Alteration of neuronal maturation. Increased. GFAP levels by 40% after 72h of injection.		↓ reelin expression
		Harvey and Boksa, 2012	Mouse	Male and female	P14, P28		Mouse offspring. Maternal injection of 0,2 mg/kg at E9.	Immunohistochemistry (NeuN, reelin and GAD67). Body weight control.	Increased neuronal density at P14. Increased GAD67 expression in ventral stratum oriens at P28 in males. Weight gain differences.		No significant differences
		Nouel et al., 2012	Rat	Male	P14, P28		Rat injected with 100 ug/kg at E15 and E16.	Cell count of GAD67- andreelin-immunoreactive neurons in HP. Western blot (reelin and GAD67).	Reduced GAD67-positive cells in dentate gyrus and CA1. Decreased reelin-positives cells in dentate gyrus and CA1.		↓ reelin positives cells in HP

*Poly(I:C), polyinosinic:polycytidylic acid; LPS, lipopolysaccharide.*

##### Influenza

This murine model consists of infecting pregnant dams with the human influenza virus (H1N1) on different days of prenatal development. This infection triggers alterations in signaling systems similar to those found in patients with SCZ (Kȩpińska et al., 2020).

Fatemi et al. (2017) studied the cerebellum (prenatal infection at E16), since this region participates in learning, memory, and emotional processing, and found a significantly lower VLDLR levels in postnatal day 14 (P14) and higher levels in young adult mice (P56). VLDLR is one of the RELN receptors, and therefore, alterations on this protein might cause impaired RELN signaling pathways and functions. They also described an increased expression of *dab1*, a downstream RELN signaling molecule, in the hippocampus (Fatemi et al., 2017). Only male mice were analyzed in this study.

##### Polyinosinic-Polycytidilic Acid

Polyinosinic-polycytidilic acid is a synthetic analog of double-stranded RNA that triggers a cytokine-associated immune response (Harvey and Boksa, 2012; Ratnayake et al., 2012; Giother with the Viravanoli et al., 2014). This compound causes an increase in the pro-inflammatory IL-6 cytokine measured 3 h after injection in maternal serum. The weight gain of pregnant females tends to differ from controls after injection (Harvey and Boksa, 2012). The phenotypes depend on both day of injection and day of dose. The mouse strain used may also influence this variable.

Infections in early (gestational day 9 (GD9)) and late (GD20) prenatal ages were coherent with the effect of Poly(I:C) on the number of RELN-positive cells. Harvey and Boksa (2012) found a reduction in the density of RELN-positive neurons in the CA1 dorsal stratum oriens at P28 compared to controls (12% decrease in males and 22% in females at GD9). Ratnayake et al. (2012) reported significantly fewer of these neurons in the hippocampus, the motor cortex, and the cerebellum at P1, and this decrease was also significant at P100 in the hippocampus at GD20 in male and female mice. Giother with the Viravanoli et al. (2014) combined prenatal immune challenge (GD9) and peripubertal stress, and they also reported a reduced number of RELN-positive cells in the hippocampus of male mice at P70. Other phenotypes described are an increase in the density of glutamate decarboxylase (GAD) 67-positive neurons in the dorsal and ventral stratum oriens in female mice (Harvey and Boksa, 2012), increased GFAP (flial fibrillary acidic protein) expression, and more activated microglia, especially in the hippocampus (Ratnayake et al., 2012).

Polyinosinic-polycytidilic acid models may show behavioral defects. Ratnayake et al. (2012) observed less distance traveled in the open field test, as well as defects in non-spatial memory (decreased latency to fall in the rotarod test) and learning tasks (decreased discrimination index in the novel object recognition test) in both sexes compared to controls.

##### Lipopolysaccharide

Infection by the bacterial endotoxin, LPS, increases the expression of pro-inflammatory cytokines like IL-6 and IL-1β in the animal (placenta, amniotic fluid, plasma, the liver, and the brain) (Ghiani et al., 2011; Harvey and Boksa, 2012). As in other models, the study of day of infection GD9 (Harvey and Boksa, 2012), GD15 and 16 (Ghiani et al., 2011; Nouel et al., 2012), sex, and age generated a wide variability of results. One of the factors commonly affected is the weight of both the injected pregnant dams (less weight gain 3 h after injection; Harvey and Boksa, 2012) and male offspring (less weight at P0 and P28; Nouel et al., 2012).

Reduced expression of RELN in the Cajal-Retzius cells of GD18 rat fetuses (Ghiani et al., 2011) and decreased number of RELN-positive neurons in the dentate gyrus and CA1 regions at P14 male rats (Nouel et al., 2012) have been described. Ghiani et al. (2011) consistently found a significant 30% decrease in 180-kDa-form RELN protein levels. This suggests a relationship between prenatal inflammation and RELN cleavage (Ghiani et al., 2011). Nouel et al. (2012) did not find changes in RELN expression, possibly because immunoreactive cell counting is a more sensitive method than a western blot. The early infection model included here did not show any alteration of RELN (Harvey and Boksa, 2012).

GAD67 alterations are controversial. The early infection model at GD9 (Harvey and Boksa, 2012) showed increased GAD67 expression and more positive neurons in the ventral stratum oriens in P28 male mice (differences in females were not found), whereas the late infection model at GD15 and 16 (Nouel et al., 2012) showed fewer GAD67-positive cells in the dentate gyrus in P14 male rats. Regarding the brain structure, Ghiani et al. (2011) found significant enlargement of the cortical plate in the neocortex of the fetuses and altered expression pattern of immature neuronal markers. Harvey and Boksa (2012) reported an increase in neuronal density in CA1 in pups.

#### Stress Models

Several studies have demonstrated the impact of the environment on brain development in prenatal and postnatal stages, and this gene-environment relationship may be one of the causes of SCZ (Matrisciano et al., 2013; Gomes et al., 2019). One of these environmental factors is stress. These SCZ models are grouped below by the time of exposure to stress, either prenatal through prenatal restraint stress (PRS) or postnatal through social isolation or maternal deprivation (Table 3).

**TABLE 3:** Principal findings of stress model studies included in this review.

	Animal model	Author	Specie	Sex	Age	Behavior characterization	Methods	Analysis	Results	Rescue experiments	Reelin alteration
Stress models	Maternal deprivation	Aksic et al., 2021	Rat	Male and female	P60		Rat offsprings with maternal deprivation from P9 to P10.	Immunohistochemistry (PV and reelin). Dell death assay.	Reduced number of PV positive interneurons in CA1 (HP) and PFC. Reduced number of reelin positive interneurons in CA1 and CA3 (HP), but unaltered in neocortex. No differences in cell death.		↓ reelin positives cells in HP
	Social isolation	Ko et al., 2016	Rat	Male and female	3 and 7-week-old	locomotion and PPI	Rat offspring isolated at P21 and mated with social rats. Offsprings of 2 next generation evaluated.	qPCR (monoamines and schizophrenia-related genes in mPFC and HP). Behavioral characterization.	Impaired PPI in second generation of rats. Lower levels of dopamine and serotonine in mPFC and HP in the third generation. Female isolation rats presented elevated reelin gene expression levels in PFC with respect to non-isolation rats in the third generation.		↑ reelin gene expression levels in PFC
	PRS	Matrisciano et al., 2013	Mouse	Male	P1, p7, p14, p60	open field, social behavior, PPI and fear conditioning	Mouse offspring exposed to PRS (30 min, 2 times per day, from E7 to E21)	RT-PCR (DNMT1 and DNMT3a of PFC and HP). Western blot (DNMT1, reelin and GAD67). Behavioral characterization.	Increased mRNA levels of DNMT1 and DNMT3a (P1 to p60) in the frontal cortex and HP. DNMT overexpression was associated with a decreased in reelin protein expression in frontal cortex. Hyperactivity and impairment of social interaction, prepulse inhibition and fear conditioning, corrected with the administration of valproic acid or clozapine.	Clozapine, Valproic acid	↓ reelin expression in frontal cortex. ↓ reelin promoter methylation
		Palacios-García et al., 2015	Rat	Male and female	E20, P60	open field, elevated plus maze, passive avoidance, Morris water maze (P60)	Rat offspring exposed to PRS (2 h, 1 time per day).	Immunohistochemistry (reelin and NeuN). Cell count of reelin- and NeuN-immunoreactive neurons. Western blot (reelin, Dab1, CDK5, PHF-1 and tubuline). Behavior characterization.	Decreased density of reelin- positive cells in cortex. Decreased reelin protein and gene expression. 79% decrease in reelin immunoreactivity in prefrontal cortex. Increased DNA methylation levels of Reelin promoter. Increased Dab1 adapter protein and decreased phosphorylation of H1 histone. Excessive spontaneous locomotor activity, high anxiety levels and impaired learning and memory consolidation.		↓ reelin positives cells in cortex. ↓ reelin expression. ↑ reelin promoter methylation
		Dong et al., 2016	Mouse	Male	P75	open field, three-chamber (P75)	Mouse offspring exposed to PRS (45 min, 3 times per day, from E7 to E21) treated with clozapine (5 mg/kg, twice a day for 5 days).	Behavior characterization. RT-qPCR (Nse, NeuN). Western blot (DNMT1). Methylated DNA immunoprecipitation.	Clozapine and valproic acid, but not haloperidol, correct the behavioral impairments in PRS mice. Clozapine and valproic acid, but not haloperidol, hypermethylation of psychiatric disorder-related genes. Clozapine treatment corrected the elevated DNMT1 protein expression level and reduced the DNMT1 binding to reelin promoter.	Clozapine, Valproic acid	↓ reelin expression in frontal cortex. ↑ reelin promoter methylation

*PRS, prenatal restraint stress.*

##### Maternal Deprivation

Aksic et al. (2021) studied the effects of maternal deprivation in the expression of neocortex and hippocampus markers in P60 male and female rats. Maternal deprivation models show usually impaired declarative memory and sensorimotor gating defects. Aksic et al. (2021) reported a reduced number of RELN positive interneurons in the CA1 and CA3 regions of the hippocampus, but not in the neocortex. These anomalies were not caused by cell death, suggesting that maternal deprivation provokes a downregulation of RELN in offspring.

##### Social Isolation

Only one study by Ko et al. (2016) on postnatal isolation after maternal weaning (P21) is included here. Social isolation models are usually hyperactive, show an enhanced response to novelty, anxiety, and aggression, and have an impaired PPI (7-week-old) (Ko et al., 2016; Winship et al., 2019). Although Ko et al. (2016) studied *reln* gene expression, they did not report significant differences regarding *reln* in male and female rats. Female isolation rats presented elevated levels of *reln* expression with respect to non-isolation rats.

##### Prenatal Restraint Stress

Prenatal restraint stress has been shown to predispose offspring to defects similar to those found in patients with SCZ, like reduced dendritic spines density in the hippocampus and changes in gene promoters’ methylation (Holloway et al., 2013). Stress in prenatal stages increases cortisol levels in the mother and male and female offspring and affects neurodevelopment (Palacios-García et al., 2015).

In E20 male and female rats, this model has shown a lower density of RELN-positive neurons in the cortex (layer I) and reduced RELN protein and gene expression. There was a 79% decrease in RELN immunoreactivity in PFC. Increased Dab1 adapter protein levels and lower phosphorylation of H1 histone, both related to the RELN pathway, have been observed (Palacios-García et al., 2015). Previous studies have reported changes in *reln* promoter methylation, specifically, increased methylation of the CpG regions. This increased methylation pattern was observed in the model (Palacios-García et al., 2015). In male mice, this model also has shown high *dnmt1* and *dnmt3a* (DNA methyltransferase 1 and 3a) expression levels in the frontal cortex and the hippocampus at birth and adulthood (P60), as well as an increased binding of DNMT1 to *reln* promoter. DNMT overexpression in GABAergic neurons was associated with a 54% decrease in RELN expression in the frontal cortex (Matrisciano et al., 2013). Clozapine treatment corrected the elevated DNMT1 expression levels and reduced the DNMT1 binding to *reln* promoter in adult male mice (Dong et al., 2016).

These adult rodent models (P60) showed increased spontaneous locomotor activity (Matrisciano et al., 2013; Palacios-García et al., 2015), decreased social interaction with a novel mouse, a deficit in PPI of startle and fear conditioning (decreased fear conditioning response and no extinction) (Matrisciano et al., 2013). They also showed high levels of anxiety (decreased time spent in the open arms of the elevated plus-maze) and impaired learning and memory consolidation in the passive avoidance test (reduced latency time in PRS rats) (Palacios-García et al., 2015). The behavioral phenotypes were corrected with clozapine and valproic acid (P75) (Matrisciano et al., 2013; Dong et al., 2016). Clozapine and valproic acid corrected the increased locomotion, social interaction deficits, and PPI defects in PRS mice (Matrisciano et al., 2013; Dong et al., 2016).

#### Genetic Models

In addition to environmental alterations that can trigger SCZ, genetic factors, such as those in the models reviewed below, showing altered RELN expression, or transgenic models of modified pathways/genes related to this protein, must also be considered (Howes et al., 2004; Nullmeier et al., 2011; Teixeira et al., 2011; Howell and Pillai, 2016; Cleynen et al., 2020; Hannon et al., 2021; Table 4).

**TABLE 4:** Principal findings of genetic model studies included in this review.

	Animal model	Author	Specie	Sex	Age	Behavior characterization	Methods	Analysis	Results	Rescue experiments	Reelin alteration
Genetic models	HRM	Nullmeier et al., 2011	Mouse	Male and female	28-week-old		HRM mouse	Immunocytochemistry (GAD67, PV, TH, 5-HT-T). Cell count and area measurement.	Decreased of GAD67-positive cells in HP and PV-positive cells in CA1 and CA2. Impairment of hippocampal GABAergic functioning.		50% reelin expression
		Teixeira et al., 2011	Mouse	Male and female	Not specified	Open field, novelty suppressed feeding, forced swim test and PPI.	HRM mouse	Behavioral characterization with or without corticosterone, cocaine sensitization and PPI with NMDA antagonist treatment.	No differences between groups in different analysis.		50% reelin expression
		van den Buuse et al., 2012	Mouse	Male and female	10–12-week-old	locomotor hyperactivity, PPI	HRM mouse	Western blot (NMDAr subunits). Behavior characterization.	Increased MK-801-induced locomotor hyperactivity in males. Altered startle reflex caused by effect of MK-801. Upregulation of NR1 subunits and down-regulation of NR2C subunits in frontal cortex.		50% reelin expression
		Romano et al., 2013	Mouse	Male	P35	Locomotion (P35)	HRM mouse injected with nicotine (1 mg/kg for 6 days)	RT-PCR (reelin, GAD67 and BDNF). Behavior characterization and its responses to nicotine.	Decreased reelin and GAD67 gene expression in PFC, HP, cerebellum and striatum. Hyperactivity in HRM. Reversion of phenotypes in HRM treated with nicotine. Increased BDNF gene expression of HRM and HRM treated with nicotine.	Nicotine	50% reelin expression
		Rogers et al., 2013	Mouse	Male and female	Not specified	Fear conditioning and PPI	HRM mouse injected with reelin	Golgi staining and morphology studies. Immunohistochemistry (GAD67). Brain slices electrophysiology. Behavioral characterization.	Reduced dendritic spine density and synaptic plasticity. Impaired behavior (associative learning and memory, PPI) in HRM. Increased GAD67 expression and altered dendritic spine morphology in HRM injected with Reelin.	Reelin injection	50% reelin expression
		Hill et al., 2013	Mouse	Male and female	Not specified		HRM mouse	Western blot (BDNF, TrkB, fosforilated TrkB and MAPK).	Increased BDNF levels in female HP and decreased phosphorylated ERK1 levels. Ovariectomy decreases BDNF expression in HP.		50% reelin expression
		Howell and Pillai, 2014	Mouse	Not specified	E18, 1 month-old, 3-month-old	PPI, Y-maze, open field PFC and HP volume.	HRM mouse with prenatal hypoxia (9% oxygen, 2 h at E17)	Levels of HIF-1a, VEGF, VEGFR2/Flk1 and GR analysis. Behavioral characterization.	Anxiety-like behavior in HRM and wt with hypoxia. Increased protein levels of VEGF in HP in HRM and wt with hypoxia. Increased protein levels of GR in Frontal Cortex in HRM and wt with hypoxia. Lower corticosterone serum levels in HRM and wt with hypoxia. Increased levels of HIF-1a and VEGF in the forebrain in HRM with hypoxia (E18). Increased GR levels in the forebrain in HRM with hypoxia (E18, 3-month-old).		50% reelin expression
		Nullmeier et al., 2014	Mouse	Male	24–28-week-old		HRM mouse	Tyrosine hydroxylase (TH)- immunoreactive and serotonin (5-HT) fibers in PFC, HP and striatum. Immunohistochemistry.	Increased tyrosine hydroxylase immunoreactive densities in HP and decreased in nucleus accumbens.		50% reelin expression
		Romano et al., 2014	Mouse	Male	P37-42	Light/dark test, openfield, hole-board test, T-maze.	HRM mouse with oral nicotine stimulation (10mg/l, from P37 to P42)	Behavior characterization. RT-PCR (Reelin, GAD67).	Nicotine restores impaired behavior (exploratory behavior and poor cognitive performance). Reelin and GAD67 mRNA expression to WT levels in PFC, HP, cerebellum and striatum.	Nicotine	50% reelin expression
		Schroeder et al., 2015	Mouse	Male and female	11-week-old	y- maze, novel object recognition, social test and PPI	HRM mouse exposed to stress (corticosterone treatment)	Reelin expression in PFC and HP. Behavioral characterization.	Increased reelin expression in PFC of female HRM treated with corticosterone. Impaired spatial memory in HRM with corticosterone. Altered PPI in male WT exposed to corticosterone, but not in male HRM.		50% reelin expression
		Mullen et al., 2016	Mouse	Male	P90		HRM mouse offspring. Prenatal infection with 20 mg/kg of CPO (GD13.5)	Western blot (reelin). Golgi staining. Nissl staining. DAPI staining.	Decreased full length and cleaved Reelin protein an altered cellular complexity and dendritic spine organization in CPO exposed mice. Reduced reelin expression by prenatal pesticide exposure can alter the shape and connectivity of neurons in several brain regions.		50% reelin expression
		Magliaro et al., 2016	Mouse	Male and female	2-month-old		HRM mouse	Number of Purkinje neurons and their topology in the cerebellar vermis	Reduced Purkinje neurons density in male and female HRM mice. Larger Purkinje neurons diameter in male HRM than female. Chaotic organization of Purkinje neurons in HRM.		50% reelin expression
		Howell and Pillai, 2016	Mouse	Not specified	6-month-old	PPI, open field and y-maze.	HRM mouse with prenatal hypoxia (9% oxygen, 2 h at E17)	Western blot (reelin, VEGF, Flk1 and GR). MRI imaging. Behavioral characterization.	Increased frontal cortex volume in prenatal hypoxia mice. Decreased frontal cortex volume in HRM with prenatal hypoxia. Decreased reelin expression in frontal cortex in prenatal hypoxic mice and HRM, and HP in HRM. Decreased HIF- 1a levels in frontal cortex and decreased serum VEGF in HRM.		50% reelin expression
		Notaras et al., 2017	Mouse	Male and female	11-week-old	Methamphetamine-induced locomotor hyperactivity, PPI, y-maze and forced swim test.	HRM mouse with oral corticosterone (50 mg/l)	Behavioral characterization.	Decreased PPI in HRM. Increased immobility in forced swim test and decreased novel arm preference in y-maze in HRM with corticosterone treatment.		50% reelin expression
	Loss of reelin function	Sakai et al., 2016	Mouse	Male	11-week-old	Open field, anxiety test, three- chamber, t-maze, Barnes maze, PPI and fear conditioning.	C-terminal reelin knock in mouse	Immunohistochemistry and western blot. Behavioral characterization. Body weight control.	Hyperactivity and reduced anxiety and social behavior. Impairment of working memory. Attenuated Reelin signaling in cortex and HP. Decreased body weight.		↓ reelin signaling in cortex and HP
		Pahle et al., 2020	Mouse	Male	15-week-old	Elevated plus maze, open field, Morris water maze.	Reelin knock out mouse (inhibitory interneurons)	Immunohistochemistry (NeuN, KI67, DCX, BrdU, GFAP, Nestin, GAD67, PV, RFP, CB1, Sox2, CCK, GFP, and Calbindin). ISH (Ndnf, Rgs8, Rorb, and Etv1). Western blot (reelin). Behavioral characterization.	Decreased reelin expression in neocortex and dentate gyrus. In dentate gyrus, increased Cajal Retzius cells number that express reelin, increased CB1 expression and decreased GFAP-positives astrocytes. NO DICE NADA DE COMPORTAMIENTO.		↓ reelin expression in neocortex and dentate gyrus
	ADAMTS KO	Ogino et al., 2017	Mouse	Not specified	E18.5		ADAMTS3 knock out mouse	ISH and immunohistochemistry (ADAMTS3 and reelin). Golgi staining. Western blot.	Recombinant ADAMTS3 cleaves Reelin at the N-t site. Decreased N-t cleavage of Reelin in ADAMTS3 KO mice. Increased dendritic branching and elongation in cortex of conditional KO mice.		↓ reelin cleavage
		Yamakage et al., 2019	Mouse	Male and female	P60		ADAMTS2 knock out mouse	qRT-PCR (ADAMTS2 and ADAMTS3). ISH (ADAMTS2).	Recombinant ADAMTS2 cleaves Reelin at the N-t site. The disintegrin domain is necessary for the Reelin-cleaving activity. ADAMTS2 is necessary for the N-t cleavage and inactivation of Reelin.		↓ reelin cleavage and reelin levels in HP
	Vldlr overexpression	Iwata et al., 2012	Rat	Not specified	2–3-month-old	Open field, locomotor activity, radial maze, social interaction and elevated plus maze.	Overexpression of Vldlr in transgenic rat	RT-qPCR and western blot (Vldlr). Histology (NeuN). Behavioral characterization.	Increased locomotor activity. Impairment of spatial working memory. Vldlr levels involved in locomotor activity and memory functions.		Altered reelin signaling (decreased Dab1 levels in PFC and cerebellum)
	Reelin overexpression	Teixeira et al., 2011	Mouse	Male and female	>8 week-old	Open field, novelty suppressed feeding, forced swim test with or without corticosterone, cocaine sensitization and PPI with NMDA antagonist treatment.	Overexpression of reelin in transgenic mouse	Behavioral characterization.	Decreased floating time (forced swim test) with corticosterone treatment and reduced sensitization to cocaine. Reelin prevents deficits in PPI induced by NMDA antagonist treatment.		↑ reelin expression

*HRM, heterozygous reeler mouse; OE, overexpression.*

##### Heterozygous Reeler Mouse

This HRM model is a *reln* haploinsufficient mouse that has been proposed as a genetic model for SCZ (Nullmeier et al., 2014, 2011). HRM has fewer GAD67-positive neurons in the hippocampus of 28-week-old female mice (Nullmeier et al., 2011). They emerged from a spontaneous mutation in mice with autosomal recessive heritability (Falconer, 1951; Magliaro et al., 2016), thus enabling the consequences of partial downregulation of RELN expression to be studied (van den Buuse et al., 2012). Despite behavioral and neuroanatomical characteristics similar to those found in patients (Nullmeier et al., 2014, 2011), such as alterations of the laminar architecture of the brain (Magliaro et al., 2016), there is wide controversy about the reproducibility of behavioral results (Teixeira et al., 2011). Some authors have described PPI deficits; however, other studies have not been able to replicate them. van den Buuse et al. (2012) described an increase in MK-801-induced locomotion in male mice and altered startle reflex in both sexes (10- to 12-week-old). Rogers et al. (2013) studied this model using RELN supplementation to revert those phenotypes (reduced expression of GAD67 and PPI deficits) in male and female mice. Moreover, reduced reelin exposure by prenatal pesticide exposure can alter the shape and connectivity of neurons in several brain regions as they have shown with HRM (male HRM at P90 with chlorpyrifos oxon exposure at GD13.5) (Mullen et al., 2016).

The NMDAr levels were altered [upregulation of NR1 subunits and downregulation of NR2C subunits in the frontal cortex (van den Buuse et al., 2012)], and defects appeared in the Brain-Derived Neurotrophic Factor (BDNF)-tropomyosin receptor kinase B (TrkB) signaling pathway (increased BDNF levels and decreased phosphorylated TrkB in the ventral hippocampus of female mice), as also described in the postmortem brains of patients (Hill et al., 2013).

The cerebellar structure of this model has also been characterized. Magliaro et al. (2016) studied the number and topology of Purkinje neurons, observing a reduction in the density of these neurons in both sexes (2 months old), and male mice, in particular, had larger, chaotically arranged Purkinje neurons.

The effects of stressors or alterations in this model have been widely studied (Rogers et al., 2013; Romano et al., 2013; Howell and Pillai, 2014, 2016; Schroeder et al., 2015; Mullen et al., 2016; Notaras et al., 2017). One such alteration is the exposure of the HRM model to prenatal hypoxia, an environmental factor closely related to SCZ (Howell and Pillai, 2014). Howell and Pillai (2014) described alterations caused by prenatal hypoxia [anxiety-like behavior at 3 months old, increased protein levels of vascular endothelial growth factor (VEGF) in the hippocampus, increased glucocorticoid receptor (GR) levels in the frontal cortex (1 month old), lower corticosterone levels at 3 months old, etc.], regardless of genotype. They also described increased levels of HIF-1α and VEGF in the forebrain at E18 and increased GR levels in the forebrain at E18 and 3 months of age in HRM. A similar study with 6-month-old mice evaluating the long-term effects of prenatal hypoxia described significant changes specific to the HRM model, such as decreased frontal cortex volume, decreased RELN expression in the hippocampus, HIF-1α levels in the frontal cortex and serum VEGF (Howell and Pillai, 2016).

In another vein, Romano et al. (2013, 2014) studied the possible short- and long-term benefits of nicotine administration to the HRM model, with different doses and administration routes (1 mg/kg-subcutaneous or 1 mg/ml-oral). In both cases, male mice had behavioral defects (hyperlocomotion) that reverted to control levels after nicotine administration (P35, Romano et al., 2013; P37-42, Romano et al., 2014). They also described the reduction of RELN and GAD67 mRNA expression in the PFC, the hippocampus, the cerebellum, and the striatum. This phenotype also reverted to normal levels after nicotine administration (Romano et al., 2014, 2013).

Two other authors studied the effects of stress caused by administering corticosterone to HRM mice (Schroeder et al., 2015; Notaras et al., 2017). Despite both studies concluding that subjecting HRM to chronic stress caused deficits in spatial memory (decreased preference for the novel arm), they differed with regard to stress defects in PPI in 11-week-old male and female mice. Schroeder et al. (2015) detected an increase in the RELN expression in the PFC of female HRM treated with corticosterone and deficits in PPI in male controls (not HRM). However, Notaras et al. (2017) described decreased PPI in HRM treated with corticosterone.

##### Loss of Reelin Function

In these two included models (Sakai et al., 2016; Pahle et al., 2020), partial loss of *reln* function is caused in the animal to study its behavioral and biochemical consequences. This loss of function can be accomplished by cleavage of a RELN region, such as the C-terminal region (Sakai et al., 2016), or by selective inactivation of its function in a certain cell type, such as inhibitory interneurons (Pahle et al., 2020).

Sakai et al. (2016) found that the C-terminal region is necessary for some RELN functions and activation of RELN downstream signaling in 11-week-old male mice. Brain structure was altered (reduced cortex layer I, CA1 pyramidal cell layer split in two, reduced granule cell layer density of dentate gyrus). RELN signaling was also reduced in the cortex and the hippocampus (a significant increase of Dab1 protein in these regions) (Sakai et al., 2016). The inhibitory interneuron knockout model demonstrated, in 15-week-old male mice, that, although RELN in these cells does not participate in adult neurogenesis or neuronal layering, its alteration has consequences for total RELN expression in the neocortex and dentate gyrus (Pahle et al., 2020). This model showed a significant reduction of RELN in these regions (35% in the dentate gyrus, 47% in the hippocampus, and 94% in the neocortex) (Pahle et al., 2020). These partial losses of RELN function can affect model behavior. C-terminal region cleavage makes the mouse hyperactive, less anxious, and less social and impairs working memory (Sakai et al., 2016), while the lack of RELN expression in inhibitory interneurons has no behavioral effect (Pahle et al., 2020).

##### A Disintegrin and Metalloproteinase With Thrombospondin Motifs Knock Out

Reelin is cleaved at three sites during processing (Yamakage et al., 2019; Jossin, 2020). One of these proteolytic cleavages takes place at the N-terminal site, which reduces RELN ability to induce Dab1 phosphorylation and inactivates RELN (Yamakage et al., 2019). Ogino et al. (2017) and Yamakage et al. (2019) studied the protease mediating this cleavage.

Reelin N-t-cleavage is mediated by secreted metalloprotease ADAMTS (a disintegrin and metalloprotease with thrombospondin motifs). This family of 19 members has a basic structure formed by a signal peptide, prodomain, metalloprotease domain, disintegrin domain, thrombospondin type 1 motifs, cysteine-rich domain, spacer domain, and ancillary domains (Yamakage et al., 2019).

Several ADAMTS have been reported to carry out the proteolytic cleavage of RELN, depending on the developmental stage. In the prenatal stages, this cleavage is mediated by ADAMTS3 in the embryonic brain, specifically in the cortex and the hippocampus. This metalloprotease is expressed in excitatory neurons in these regions. There is less RELN inactivation and lower Dab1 levels and phosphorylated Tau in the cortex of the mouse model that does not express this metalloprotease (E18.5 ADAMTS3 Knock out). Dab1 and phosphorylation Tau levels inversely correlate with RELN activity (Ogino et al., 2017).

In the postnatal stages, ADAMTS2 mediates RELN N-t-site cleavage in the cortex and the hippocampus of the postnatal brain just as efficiently as ADAMTS3 in the prenatal stages. The disintegrin domain is required for ADAMTS2 to process RELN correctly. This was confirmed by generating an ADAMTS2 knockout model that showed lower RELN levels in the cerebral cortex and the hippocampus in P60 male and female mice (Yamakage et al., 2019). Recent studies have found ADAMTS2 upregulation in patients with SCZ at basal conditions restored to control levels following antipsychotic treatment. This provides further support for the relationship between *ADAMTS2* and *RELN* and points to *ADAMTS2* upregulation as the possible cause of the reduced *RELN* expression in patients with SCZ (Sainz et al., 2013; Crespo-Facorro et al., 2014; Ruso-Julve et al., 2019).

##### Very-Low Density Lipoprotein Receptor Overexpression

The VLDLR and low-density lipoprotein receptor-related protein 8 (Lrp8), as well as the intracellular adapter protein Dab1, are a part of the RELN signaling pathway (Iwata et al., 2012; Jossin, 2020). Following studies that reported increased VLDLR mRNA levels in the brain of patients with autism, Iwata et al. (2012) generated a rat model that overexpressed *vldlr* in the brain. RELN binds to VLDLR and Lrp8 to regulate neuronal layering positioning in cortical brain regions. Although these rats do not have any morphological brain abnormalities, they do show behavioral alterations, such as increased spontaneous locomotor activity and defects in working memory, as evaluated in the radial maze. RELN binding to VLDLR triggers the phosphorylation of Dab1. Dab1 has been found to be reduced in patients with autism. In this model, Iwata et al. (2012) reported decreased *dab1* expression in the PFC and the cerebellum, showing that VLDLR overexpression alters downstream RELN signaling (2- to 3-month-old rats) (Iwata et al., 2012).

##### Reelin Overexpression

Few studies have focused on the role of RELN as a treatment for SCZ. Teixeira et al. (2011) generated a mouse model that overexpresses RELN to evaluate the direct influence of RELN levels on mouse behavior. Under basal conditions, this model does not have behavior deficits. However, RELN overexpression mice (>8-week-old) do show defects after corticosterone treatment. Wild-type mice with corticosterone spent significantly more time floating in the forced swim test than RELN overexpression mice and control mice. This suggests that RELN overexpression mice were less sensitive to helplessness-like behavior induced by corticosterone. RELN overexpression mice also showed defects after cocaine (reduced sensitization to continued cocaine administration) or NMDA antagonist (prevents impaired PPI) treatments. This model demonstrated that RELN overexpression in the mouse forebrain protects against environmental stressors that are related to the cause of psychiatric disorders such as SCZ (Teixeira et al., 2011).

## Discussion

Our main findings show that SCZ rodent models (pharmacological models, MIA, stress, and genetic models) can be categorized depending on the type of RELN alteration described: reduced RELN expression, fewer RELN-positive cells, alteration of RELN signaling, and different methylation patterns. The fact that RELN is altered in all the models, despite their diversity, shows that the RELN pathway is one of the keys to SCZ disease and a preclinical biomarker common to all models.

This review helps clarify the differences and variability in results between models and between authors by schematically presenting the main findings of each study, highlighting the multifactorial nature of SCZ models. Even though all models were of the same disease, not all authors agreed on the main symptoms or on how RELN function and expression are altered. Patients with SCZ have been reported to have lower RELN levels in the brain (Brosda et al., 2011; Nullmeier et al., 2011; Schroeder et al., 2015). Some of the studies included in this review found lower RELN expression (Brosda et al., 2011; Ghiani et al., 2011; Matrisciano et al., 2013; Palacios-García et al., 2015; Dong et al., 2016; Yamakage et al., 2019; Pahle et al., 2020), a decrease in the number of RELN-positive cells (Harvey and Boksa, 2012; Nouel et al., 2012; Ratnayake et al., 2012; Radonjić et al., 2013; Giother with the Viravanoli et al., 2014; Palacios-García et al., 2015; Aksic et al., 2021), altered RELN signaling (Iwata et al., 2012; Sakai et al., 2016; Fatemi et al., 2017; Ogino et al., 2017; Yamakage et al., 2019), or a different methylation pattern (Matricon et al., 2010; Matrisciano et al., 2013; Palacios-García et al., 2015; Dong et al., 2016). Very few models did not find direct RELN alterations (Harvey and Boksa, 2012; Ishii et al., 2015b; Ko et al., 2016; Table 5). All the studies in HRM models corroborated 50% RELN expression (Nullmeier et al., 2011, 2014; Teixeira et al., 2011; van den Buuse et al., 2012; Hill et al., 2013; Rogers et al., 2013; Romano et al., 2013, 2014; Schroeder et al., 2015; Howell and Pillai, 2016; Magliaro et al., 2016; Mullen et al., 2016; Notaras et al., 2017). Despite the fact that each author focused on a different region of the brain, most of the alterations were described in the PFC and the hippocampus, being both regions highly involved in SCZ disease. The only three models that overexpress RELN instead of reducing levels are the Teixeira et al. (2011) transgenic model, in which they caused this overexpression to study its consequences in mice, the RELN injections models (7 to 9-week-old male mice, Sawahata et al., 2021; 6 to 7-week-old male mice, Ishii et al., 2015a) and the high-fat diet model (12 to 24-week-old mice) (Roberts et al., 2019; Table 5). A recent study supports the hypothesis of RELN as a treatment for SCZ. It reported that RELN microinjection in the hippocampus of an MIA model mouse can rescue deficits in memory (novel object recognition) and anxiety-like behavior (Ibi et al., 2020).

**TABLE 5:** Principal findings of other model studies included in this review.

	Animal model	Author	Specie	Sex	Age	Behavior characterization	Methods	Analysis	Results	Rescue experiments	Reelin alteration
Other models	Reelin injection	Ishii et al., 2015a	Mouse	Male	6–7-week-old	PPI, novel object recognition.	Reelin injection in lateral ventricle of mouse and PCP (1 mg/kg) 30 min before behavior test	Immunohistochemistry (VLDLR, CaMKII and PV). Behavioral characterization.	Reelin injection prevents PCP- induced behavioral phenotypes, increases dendritic spines density and synaptic plasticity, and improves memory and spatial learning.	Reelin injection	Reelin injection
		Ishii et al., 2015b	Mouse	Male	P14.5, 7-month-old	Y- maze, three chamber test (P14.5)	Mice with focal heterotopias in somatosensory cortex	*In situ* hybridation. Behavioral characterization.	Impaired spatial working memory and low competitive dominant behavior. Decreased immediate early gene expression.		No differences
		Sawahata et al., 2021	Mouse	Male	7–9-week-old	NOR, PPI, y-maze.	Reelin injection in mPFC of MK-801 model (0,15 mg/kg 30 min before behavior test).	Immunohistochemistry (c-Fos positive cells in mPFC). Behavioral characterization.	Reelin injection prevents MK- 801-induced impairment of recognition memory. No effect of treatment in sensory-motor gating or short term memory defects. Reelin treatment reduced number of c-Fos positive cells to control levels in mPFC.	Reelin injection	Reelin injection
	High fat diet	Roberts et al., 2019	Mouse	Male	12–24-week-old		Mouse with high fat diet (12–16 weeks)	qPCR and western blot (ApoER2 and VLDLR). ISH (RELN, VLDLR and ApoER2).	Reduced expression of ApoER2 and VLDLR and increased levels of reelin protein in hypothalamus.		↑ reelin expression
	Reelin antisense	Brosda et al., 2011	Rat	Male	P43, p93	PPI, startle reflex, working memory, novel object recognition, locomotor activity.	Reelin antisense (knock down) in PFC of rats	Western blot (reelin in mPFC). Behavioral characterization.	Impairment of PPI, spatial memory and novel object recognition. Selective alteration of mPFC. Low reelin protein expression		↓ reelin expression

During postnatal development and adult stages, *RELN* is expressed in GABAergic interneurons and modulates NMDA receptor activity and synaptic plasticity (Harvey and Boksa, 2012). GAD67 is a marker for γ-aminobutyric acid (GABA) neurons and is responsible for converting glutamate to GABA (Harvey and Boksa, 2012). Changes in GABAergic neurotransmission and synaptic plasticity could result in a brain vulnerable to other environmental and genetic risk factors, and the interaction of these factors could give rise to SCZ (Harvey and Boksa, 2012). *RELN*, *GAD65*, and *GAD67* levels are also often altered in patients with SCZ (postmortem studies) (Nullmeier et al., 2011; de Jonge et al., 2017). In the articles reviewed, these results were also contradictory. Some models showed higher levels of GAD65/67 (Harvey and Boksa, 2012; Rogers et al., 2013; Fatemi et al., 2017), while others described lower levels (Nullmeier et al., 2011; Nouel et al., 2012; Matrisciano et al., 2013; Romano et al., 2013, 2014) depending on the brain area studied and the model. Further studies are needed to determine the reason for this variability in results, even within the same type of model.

Similar diversity was found in the behavioral differences between models and their controls. The variability found in the open field test and prepulse inhibition analysis revealed the heterogeneity of these models, which is why further studies are necessary to help establish better defined, more comparable protocols. Other behavior tests evaluated memory (spatial and object recognition associated with fear) and sociability, but no overall statistical analysis was possible due to the small sample sizes. The variability in the results on RELN alteration in rodent models of SCZ reviewed here highlights the need to agree on model protocols.

Our results support the hypothesis of genetic and environmental causes of SCZ disease. Various alterations of environmental factors during prenatal and early postnatal development (stress, viral and bacterial infections, drug exposure) have been reported to cause symptoms similar to those described in patients with SCZ. Roberts et al. (2019) studied the consequences of a high-fat diet on RELN levels and its main receptors (ApoER2 and VLDLR) in a mouse model and concluded that this protein is affected by diet-induced obesity. Patients with SCZ commonly have metabolism and obesity issues. In this regard, more research on diet in SCZ models is necessary, as a high-fat diet has been found to alter RELN expression in areas of the brain related to this disease (Roberts et al., 2019). Several authors have also described differences in model weight gain. In PRS models, a 10–15% smaller weight gain than in controls was observed in mice (Matrisciano et al., 2013) and 22.8% in rats (Palacios-García et al., 2015), while the Harvey and Boksa (2012) LPS model weighed more than controls at birth. The adult models may also differ in weight. Harvey and Boksa (2012) described a lower weight in two MIA, LPS, and Poly (I:C) mouse models. Another LPS model corroborated these results (Nouel et al., 2012). The loss of *reln* function model (Sakai et al., 2016) also showed a decrease in mean body weight.

Another environmental factor reported in this review as a cause of SCZ is prenatal neuroinflammation. The articles included here studied pro-inflammatory cytokines and glial alterations (microglia and astroglia), especially in MIA models (Ghiani et al., 2011; Harvey and Boksa, 2012; Ratnayake et al., 2012). It would be of interest for the possible alterations in neuroinflammation markers to be evaluated in other types of SCZ models. Finally, it is worth mentioning the influence of sex in this clearly sexually dimorphic disease, in which men and women do not always present the same symptoms or outcome (Mendrek and Stip, 2011; Ayesa-Arriola et al., 2020). Until recently, most studies in rodent models have only used males to avoid variability in results caused by hormonal cycles in females. However, in recent years, females have begun to be included in many studies, and their results vary between sexes. Harvey and Boksa (2012) found a reduction in the density of RELN-positive neurons in the CA1 dorsal stratum oriens at P28 compared to controls (12% decrease in males and 22% in females at GD9) in the Poly (I:C) model. Other articles included in this review do not specify whether the differences found in RELN are sex-dependent. Therefore, it remains unclear whether there is a sexually dimorphic expression of RELN in the brain of these rodent models of SCZ. Harvey and Boksa (2012) described increased GAD67 expression in ventral stratum oriens specifically in female mice injected with Poly (I:C) and in male mice injected with LPS. van den Buuse et al. (2012) only observed MK-801-induced hyperlocomotion in HRM male mice and the same group found increased BDNF levels in HRM hippocampus of female mice (Hill et al., 2013). Magliaro et al. (2016) reported larger Purkinje neurons in male HRM. Future studies with rodent models of this disease should include both female and male rodent models in all analyzed parameters.

This review has some limitations. First, many of the rodent models described are not only specific to SCZ. For example, MIA models are often used to mimic autism symptoms, and stress models are usually associated with depression. However, we tried to minimize this limitation by specifically selecting studies that mention SCZ and were centered on positive, negative, and cognitive symptoms associated with this psychiatric disorder. Second, the search is limited to studies that include RELN analysis. Therefore, since not all existing SCZ rodent models are included in our systematic review, we cannot rule out whether RELN could also be altered in those models.

## Conclusion and Future Directions

Our results conclude that RELN is an altered biomarker common to all included rodent models, mainly those that involve prenatal or genetic alterations, and a key protein of SCZ, making the RELN signaling pathway in prenatal stages a target of special interest for future preclinical and clinical studies. We have shown that all different rodent models have at least one of the four described types of RELN alterations, thus highlighting this protein as a common substrate that will contribute to the face validity of future SCZ models. Future study designs should include both female and male animals due to the sexual dimorphism of SCZ, and behavioral evaluation protocols are necessary to minimize heterogeneity of results.

## Data Availability Statement

The original contributions presented in the study are included in the article/Supplementary Material, further inquiries can be directed to the corresponding author/s.

## Author Contributions

ACS-H and CM-C wrote the first draft of the manuscript. ACS-H, CM-C, and NG-T did the quality assessment of the included studies. NG-T managed the literature searches and wrote the protocol. BC-F revised the manuscript critically. All authors contributed to the first draft of the manuscript and have revised and approved the final manuscript.

## Conflict of Interest

The authors declare that the research was conducted in the absence of any commercial or financial relationships that could be construed as a potential conflict of interest.

## Publisher’s Note

All claims expressed in this article are solely those of the authors and do not necessarily represent those of their affiliated organizations, or those of the publisher, the editors and the reviewers. Any product that may be evaluated in this article, or claim that may be made by its manufacturer, is not guaranteed or endorsed by the publisher.

## Abbreviations

ADAMTS: a disintegrin and metalloproteinase with thrombospondin motifs
BDNF: brain-derived neurotrophic factor
Dab1: disabled homolog-1
DNMT: DNA methyltransferase
E: embryonic day
GAD65/67: glutamate decarboxylase 65/67
GD: gestational day
HP: hippocampus
HRM: heterozygous reeler mouse
LPS: lipopolysaccharide
MAM: methylazoxymethanol acetate
MIA: maternal immune activation
NMDAr: N-methyl -D-aspartate receptor
P: postnatal day
PCP: phencyclidine
PFC: prefrontal cortex
Poly(I:C): polyinosinic:polycytidylic acid
PPI: prepulse inhibition
PRS: prenatal restraint stress
RELN: reelin
VLDLR: very-low density lipoprotein receptor.
