# Supplementary material for: Reelin Alterations, Behavioral Phenotypes, and Brain Anomalies in Schizophrenia: A Systematic Review of Insights From Rodent Models
Source: Front Neuroanat. 2022 Mar 24;16:844737. doi: 10.3389/fnana.2022.844737 (PMC8986979; doi:10.3389/fnana.2022.844737)
Supplement: Supplementary file 1 [file Data_Sheet_1.pdf]

Reelin alterations, behavioral phenotypes and brain anomalies in schizophrenia: A systematic review of insights from rodent models.

Ana C. Sánchez-Hidalgo [1,2 †], Celia Martín-Cuevas [1,2 †], Benedicto Crespo-Facorro [1,2,3 *], Nathalia Garrido-Torres [1,2,3]

Index

Tables 2

Table S1. Search Strategy..2

Table S2. Full text of excluded articles… 2

Table S3a. Genetic model studies …...12

Table S3b. Environmental model studies………………………………………….………..14

Table S3c Gene x environmental model studies …………………………………………….. 17

Quality assessment procedures S4.. 19

Tables

Table S1. Search Strategy

**PubMed Search Query**	("psychotic disorder" OR schizophreni*) AND (reelin) AND (brain OR cerebr* OR blood OR mice OR rat OR human OR patient) NOT (genome OR polymorphism* OR review) AND full text[sb]
**Web of Science Search Query**	TOPIC:("psychotic disorder" OR schizophreni*) *AND* TOPIC:(reelin) *AND* TOPIC: (brain OR cerebr* OR blood OR mice OR rat OR human OR patient) *NOT* TOPIC: (genome OR polymorphism) *NOT* DOCUMENT TYPES: (Review)

Table S2. Full text of excluded articles

**Author**	**Title**	**Year**
Gavrilovici, C	Behavioral déficits in mice with postnatal disruption of Ndel1 in forebrain excitatory neurons: implications for epilepsy and neuropsychiatric disorders	2021
Zhang, YD	Transcription factor 4 controls positioning of cortical projections neurons through regulation of cell adhesion	2021
Nie, F	Schizophrenia risk candidate EGR3 is a novel transcriptional regulator of RELN and regulates neurite outgrowth via the reelin signal pathway in vitro	2021
Yin, J	Exploring the mRNA expression level of RELN peripheral blood of schizophrenia patients before and after antipsychotic treatment	2020
Lebedeva, KA	Cyclical administration of corticosterone results in aggravation of depression-like behaviors and accompanying downregulations in reelin in an animal model of chronic stress relevant to human recurrent depression	2020
Ho, NF	Plasticity of DNA methylation, functional brain connectivity and efficiency in cognitive remediation for schizophrenia	2020
Hume, C	The Effect of Chronic Methamphetamine Treatment on Schizophrenia Endophenotypes in Heterozygous Reelin Mice: Implications for Schizophrenia	2020
Nawa, Y	Rare single-nucleotide DAB1 variants and their contribution to Schizophrenia and autism spectrum disorder susceptibility	2020
Vazquez-Borsetti,	Deep hypothermia prevents striatal alterations produced by perinatal asphyxia: Implications for the prevention of dyskinesia and psychosis	2020
Bai, W	Decreased serum levels of reelin in patients with schizophrenia	2020
Bradshaw, NJ	Disrupted in Schizophrenia 1 regulates the processing of reelin in the perinatal cortex	2020
Khoshghadam, S	The effect of exercise on reelin level in the hippocampus of diabetic rats	2020
Armstrong, NC	Reelin: Diverse roles in central nervous system development, health and disease	2019
Dalla Vecchia E	Reelin Signaling Controls the Preference for Social Novelty in Zebrafish.	2019
Talebian, S	Assessment of expression of RELN signaling pathway in multiple sclerosis patients	2019
Vazquez-Borsetti, P	Deep hypothermia reverses behavioral and histological alterations in a rat model of perinatal asphyxia	2019
Dong, EB	N-Phthalyl-L-Tryptophan (RG108), like Clozapine (CLO), Induces Chromatin Remodeling in Brains of Prenatally Stressed Mice	2019
Sobue, A	Genetic and animal model analyses reveal the pathogenic role of a novel deletion of RELN in schizophrenia	2018

Shehabeldin, R	Reelin controls the positioning of brainstem serotonergic raphe neurons	2018
Brietzke, E	The impact of body mass index in gene expression of reelin pathway mediators in individuals with schizophrenia and mood disorders: A postmortem study	2018
Molinard-Chenu, A	The Candidate Schizophrenia Risk Gene DGCR2 Regulates Early Steps of Corticogenesis	2018
Li, TC	Proteomic study revealed antipsychotics-induced nuclear protein regulations in B35 cells are similar to the regulations in C6 cells and rat cortex	2018
Al Sagheer, T	Neurological soft signs in bipolar and unipolar disorder: A case-control study	2018
Kondratiev, N	Methylation status of the reelin gene (RELN) promoter in the blood and cognitive performance of schizophrenic patients	2018
Luoni, A	Altered expression of schizophrenia-related genes in mice lacking mGlu5 receptors	2018
Lin, L	Epigenetic regulation of reelin expression in multiple myeloma	2017
Stachowiak, EK	Cerebral organoids reveal early cortical maldevelopment in schizophrenia-computational anatomy and genomics, role of FGFR1	2017
Farag, MI	Rapgef2, a guanine nucleotide exchange factor for Rapl small GTPases, plays a crucial role in adherence junction (AJ) formation in radial glial cells through ERK-mediated upregulation of the AJ-constituent protein expression	2017
Shieu, MJ	Up-regulated microRNA MIR-34A in peripheral blood of patients with Schizophrenia and reelin as its target gene: identification from algorithms and validation in reporter gene assays	2017
Song, W	Parkinsonian features in aging GFAP.HMOX1 transgenic mice overexpressing human HO-1 in the astroglial compartment	2017
Chang, X	RNA-seq analysis of amygdala tissue reveals characteristic expression profiles in schizophrenia	2017
Antonioli-Santos, R	The fine tuning of retinocollicular topography depends on reelin signaling during early postnatal development of the rat visual system	2017
Imai, H	Dorsal Forebrain-Specific Deficiency of Reelin-Dab1 Signal Causes Behavioral Abnormalities Related to Psychiatric Disorders	2017
Varbanov, H	Regulation of extrasynaptic signaling by polysialylated NCAM: Impact for synaptic plasticity and cognitive functions	2017
Fikri, RMN	Reelin (RELN) DNA methylation in the peripheral blood of schizophrenia	2017
Tee, JY	Cell migration in schizophrenia: Patient-derived cells do not regulate motility in response to extracellular matrix	2017
Vomund, S	Behavioral Resilience and Sensitivity to Locally Restricted Cortical Migration Deficits Induced by In Utero Knockdown of Disabled-1 in the Adult Rat	2017
Serrano-Morales, JM	Reelin-Dab1 signaling system in human colorectal cancer	2017
Iafrati, J	Multivariate synaptic and behavioral profiling reveals new developmental endophenotypes in the prefrontal cortex	2016
Mizukami, T	CUB and Sushi multiple domains 3 regulates dendrite development	2016
Tee, JY	Schizophrenia patient-derived olfactory neurosphere-derived cells do not respond to extracellular reelin	2016
Hill, RA	Sex differences in animal models of schizophrenia shed light on the underlying pathophysiology	2016
Jiang, YL	Ndel1 and Reelin Maintain Postnatal CA1 Hippocampus Integrity	2016
Yamada, K	Behavioral and neurochemical abnormalities in heterozygous Reelin Orleans mutant mouse model of schizophrenia	2016
Maeta, K	Crucial Role of Rapgef2 and Rapgef6, a Family of Guanine Nucleotide Exchange Factors for Rap1 Small GTPase, in Formation of Apical Surface	2016

	Adherens Junctions and Neural Progenitor Development in the Mouse Cerebral Cortex	
Lintas, C	Differential methylation at the RELN gene promoter in temporal cortex from autistic and typically developing post-puberal subjects	2016
Davis, KN	GAD2 Alternative Transcripts in the Human Prefrontal Cortex, and in Schizophrenia and Affective Disorders	2016
Vazquez-Borsetti, P	Perinatal Asphyxia Reduces the Number of Reelin Neurons in the Prelimbic Cortex and Deteriorates Social Interaction in Rats	2016
Peedicayil, J	Preclinical epigenetic models for screening epigenetic drugs for schizophrenia	2016
Ching, AS	A Perspective on the Role of microRNA-128 Regulation in Mental and Behavioral Disorders	2015
Hornig, T	Increased Blood-Reelin-Levels in First Episode Schizophrenia (vol 10, e0134671, 2015)	2015
Zhao, LN	Ezh2 is involved in radial neuronal migration through regulating Reelin expression in cerebral cortex	2015
Hornig, T	Increased Blood-Reelin-Levels in First Episode Schizophrenia	2015
Murillo, B	Zic2 Controls the Migration of Specific Neuronal Populations in the Developing Forebrain	2015
Kigar, SL	Gadd45b is an epigenetic regulator of juvenile social behavior and alters local pro-inflammatory cytokine production in the rodent amygdala	2015
Varela, MJ	Reelin influences the expression and function of dopamine D-2 and serotonin 5-HT2A receptors: a comparative study	2015
Sibbe, M	Stem- and Progenitor Cell Proliferation in the Dentate Gyrus of the Reeler Mouse	2015
Romay-Tallon, R	Differential effects of corticosterone on the colocalization of reelin and neuronal nitric oxide synthase in the adult hippocampus in wild type and heterozygous reeler mice	2015
da Silva, VAM	Testosterone Depletion Induces Demethylation of Murine Reelin Promoter CpG Dinucleotides: A Preliminary Study	2015
Lui, CC	Effects of Melatonin on Prenatal Dexamethasone-Induced Epigenetic Alterations in Hippocampal Morphology and Reelin and Glutamic Acid Decarboxylase 67 Levels	2015
Gebicke-Haerter, PJ	The Impact of Epigenetics in Schizophrenia Research	2015
Pohlkamp, T	Characterization and Distribution of Reelin-Positive Interneuron Subtypes in the Rat Barrel Cortex	2014
Thom, M	Interictal psychosis following temporal lobe surgery: dentate gyrus pathology	2014
Camacho, J	RELN-expressing neuron density in layer I of the superior temporal lobe is similar in human brains with autism and in age-matched controls	2014
Guidotti, A	DNA methylation and demethylation as targets for antipsychotic therapy	2014
Buret, L	Corticosterone treatment during adolescence induces down-regulation of reelin and NMDA receptor subunit GLUN2C expression only in male mice: implications for schizophrenia	2014
Buret, L	Reduced expression of both brain-derived neurotrophic factor and reelin sensitizes to the long-term effects of glucocorticoid stimulation on memory in mice: relevance to schizophrenia	2014
Iafrati, J	Reelin, an extracellular matrix protein linked to early onset psychiatric diseases, drives postnatal development of the prefrontal cortex via GluN2B-NMDARs and the mTOR pathway	2014
Teixeira, CM	Transient Downregulation of Dab1 Protein Levels during Development Leads to Behavioral and Structural Deficits: Relevance for Psychiatric Disorders	2014
Kundakovic, M	DNA Methyltransferase Inhibitors and Psychiatric Disorders	2014
Yamashita, N	Mice lacking collapsin response mediator protein 1 manifest hyperactivity, impaired learning and memory, and impaired prepulse inhibition	2013

Shin, J and Kim, J	Novel alternative splice variants of chicken NPAS3 are expressed in the developing central nervous system	2013
Hoirisch-Clapauch, S	Multiple Roles of Tissue Plasminogen Activator in Schizophrenia Pathophysiology	2013
Chase, KA	Histone methylation at H3K9: Evidence for a restrictive epigenome in schizophrenia	2013
Peterson, SM	Developmental reelin expression and time point-specific alterations from lead exposure in zebrafish	2013
Parboosing, R	Gestational Influenza and Bipolar Disorder in Adult Offspring	2013
Zhao, Q	Neurological soft signs discriminate schizophrenia from major depression but not bipolar disorder	2013
Yang XB	Association study of the reelin (RELN) gene with Chinese Va schizophrenia.	2013
Swerdlow NR	Coupling of gene expression in medial prefrontal cortex and nucleus accumbens after neonatal ventral hippocampal lesions accompanies deficits in sensorimotor gating and auditory processing in rats.	2013
Rideau Batista Novais, A	N-acetyl-cysteine prevents pyramidal cell disarray and reelin-immunoreactive neuron deficiency in CA3 after prenatal immune challenge in rats	2013
Wong, J	Expression of NPAS3 in the Human Cortex and Evidence of Its Posttranscriptional Regulation by miR-17 During Development, With Implications for Schizophrenia	2013
de Lacy, N	Revisiting the Relationship Between Autism and Schizophrenia: Toward an Integrated Neurobiology	2013
Mullen, BR	Decreased reelin expression and organophosphate pesticide exposure alters mouse behaviour and brain morphology	2013
Li, AH	Lysine-specific demethylase 1 expression in zebrafish during the early stages of neuronal development	2012
Bader, V	Proteomic, genomic and translational approaches identify CRMP1 for a role in schizophrenia and its underlying traits	2012
Dong, E	Upregulation of TET1 and downregulation of APOBEC3A and APOBEC3C in the parietal cortex of psychotic patients	2012
Bonsch, D	Methylation matters? Decreased methylation status of genomic DNA in the blood of schizophrenic twins	2012
Song, W	Schizophrenia-Like Features in Transgenic Mice Overexpressing Human HO-in the Astrocytic Compartment	2012
Kimoto, S	Selective overexpression of Comt in prefrontal cortex rescues schizophrenia-like phenotypes in a mouse model of 22q11 deletion syndrome	2012
Kadriu, B	DNA methyltransferases1 (DNMT1) and 3a (DNMT3a) colocalize with GAD67-positive neurons in the GAD67-GFP mouse brain	2012
Kirkbride, JB	Prenatal nutrition, epigenetics and schizophrenia risk: can we test causal effects?	2012
Rivera-Baltanas, T	Serotonin transporter clustering in blood lymphocytes as a putative biomarker of therapeutic efficacy in major depressive disorder	2012
Berretta, S	Extracellular matrix abnormalities in schizophrenia	2012
Thanseem, I	Elevated Transcription Factor Specificity Protein 1 in Autistic Brains Alters the Expression of Autism Candidate Genes	2012
van den Buuse M	Altered N-methyl-D-aspartate receptor function in reelin heterozygous mice: male-female differences and comparison with dopaminergic activity.	2012
Zhu, Q	Increased Expression of DNA methyltransferase 1 and 3a in Human Temporal Lobe Epilepsy	2012
Habl, G	Decreased Reelin Expression in the Left Prefrontal Cortex (BA9) in Chronic Schizophrenia Patients	2012
Kobow, K	The methylation hypothesis: Do epigenetic chromatin modifications play a role in epileptogenesis?	2011
Matrisciano, F	Activation of Group II Metabotropic Glutamate Receptors Promotes DNA Demethylation in the Mouse Brain	2011

D'Aiuto, L	Mouse ES cells overexpressing DNMT1 produce abnormal neurons with upregulated NMDA/NR1 subunit	2011
Blumkin, E	Gender-Specific Effect of Mthfr Genotype and Neonatal Vigabatrin Interaction on Synaptic Proteins in Mouse Cortex	2011
Chen, F	The Structure of Neurexin 1 alpha Reveals Features Promoting a Role as Synaptic Organizer	2011
Lussier, AL	Reelin as a putative vulnerability factor for depression: Examining the depressogenic effects of repeated corticosterone in heterozygous reeler mice	2011
Chen, Y	Analysis of the GAD1 promoter: Trans-acting factors and DNA methylation converge on the 5 ' untranslated region	2011
Ovadia, G	The Genetic Variation of RELN Expression in Schizophrenia and Bipolar Disorder	2011
Senturk, A	Ephrin Bs are essential components of the Reelin pathway to regulate neuronal migration	2011
Santarelli, DM	Upregulation of Dicer and MicroRNA Expression in the Dorsolateral Prefrontal Cortex Brodmann Area 46 in Schizophrenia	2011
Grayson, DR	Epigenetic Regulation of GABAergic Targets in Psychiatry	2011
Beveridge, NJ	Schizophrenia is associated with an increase in cortical microRNA biogenesis	2010
Dong, E	Valproate induces DNA demethylation in nuclear extracts from adult mouse brain	2010
Tueting, P	L-methionine decreases dendritic spine density in mouse frontal cortex	2010
Fatemi, SH	Co-occurrence of neurodevelopmental genes in etiopathogenesis of autism and schizophrenia	2010
Host, L	Inhibition of histone deacetylases in rats self-administering cocaine regulates lissencephaly gene-1 and reelin gene expression, as revealed by microarray technique	2010
Maloku, E	Lower number of cerebellar Purkinje neurons in psychosis is associated with reduced reelin expression	2010
Rivera-Baltanas T	Serotonin transporter clustering in blood lymphocytes of reeler mice.	2010
Tseng, WL	Reelin is a platelet protein and functions as a positive regulator of platelet spreading on fibrinogen	2010
Lintas, C	Neocortical RELN promoter methylation increases significantly after puberty	2010
Kanduc, D	Describing the hexapeptide identity platform between the influenza A H5N1 and Homo sapiens proteomes	2010
Marques, SCF	Epigenetics and Neurodegeneration: A Connection Overlooked	2010
Suárez-Solá ML	Neurons in the white matter of the adult human neocortex.	2009
Fatemi SH	Chronic psychotropic drug treatment causes differential expression of Reelin signaling system in frontal cortex of rats.	2009
Kohno, T	C-Terminal Region-Dependent Change of Antibody-Binding to the Eighth Reelin Repeat Reflects the Signaling Activity of Reelin	2009
Dong, EB	Antipsychotic subtypes can be characterized by differences in their ability to modify GABAergic promoter methylation	2009
Biamonte, F	Interactions between neuroactive steroids and reelin haploinsufficiency in Purkinje cell survival	2009
Gavin, DP	Histone deacetylase inhibitors and candidate gene expression: An in vivo and in vitro approach to studying chromatin remodeling in a clinical population	2009
Ammassari-Teule, M	Reelin haploinsufficiency reduces the density of PV plus neurons in circumscribed regions of the striatum and selectively alters striatal-based behaviors	2009

Zhubi, A	An upregulation of DNA-methyltransferase 1 and 3a expressed in telencephalic GABAergic neurons of schizophrenia patients is also detected in peripheral blood lymphocytes	2009
Campo, CG	Reelin Secreted by GABAergic Neurons Regulates Glutamate Receptor Homeostasis	2009
Gavin, DP	Dimethylated lysine 9 of histone 3 is elevated in schizophrenia and exhibits a divergent response to histone deacetylase inhibitors in lymphocyte cultures	2009
Kobow, K	Increased Reelin Promoter Methylation Is Associated With Granule Cell Dispersion in Human Temporal Lobe Epilepsy	2009
Pisanté A	A variant in the reelin gene increases the risk of schizophrenia and schizoaffective disorder but not bipolar disorder.	2009
Ayhan Y	Animal models of gene-environment interactions in schizophrenia.	2009
Guidotti A	Characterization of the action of antipsychotic subtypes on valproate-induced chromatin remodeling.	2009
Kundakovic, M	The Reelin and GAD67 Promoters Are Activated by Epigenetic Drugs That Facilitate the Disruption of Local Repressor Complexes	2009
Fish, KN	Functional consequences of hippocampal neuronal ectopia in the apolipoprotein E receptor-2 knockout mouse	2008
Abdolmaleky HM	Epigenetic alterations of the dopaminergic system in major psychiatric disorders.	2008
Weiser, M	Advanced Parental Age at Birth Is Associated With Poorer Social Functioning in Adolescent Males: Shedding Light on a Core Symptom of Schizophrenia and Autism	2008
Satta, R	Nicotine decreases DNA methyltransferase 1 expression and glutamic acid decarboxylase 67 promoter methylation in GABAergic interneurons	2008
Dong, E	Clozapine and sulpiride but not haloperidol or olanzapine activate brain DNA demethylation	2008
Nudelman, A	A mutual prodrug ester of GABA and perphenazine exhibits antischizophrenic efficacy with diminished extrapyramidal effects	2008
Meyer, U	Adult brain and behavioral pathological markers of prenatal immune challenge during early/middle and late fetal development in mice	2008
Barr, AM	Heterozygous reeler mice exhibit alterations in sensorimotor gating but not presynaptic proteins	2008
Tochigi, M	Methylation status of the reelin promoter region in the brain of schizophrenic patients	2008
Pillai, A	Increased truncated TrkB receptor expression and decreased BDNF/TrkB signaling in the frontal cortex of reeler mouse model of schizophrenia	2008
Dimitriadis, EA	The Role of Reelin in the Brain Overview	2008
Sharma, RP	Histone deactylase 1 expression is increased in the prefrontal cortex of schizophrenia subjects: Analysis of the National Brain Databank microarray collection	2008
Suzuki, K	Decreased expression of reelin receptor VLDLR in peripheral lymphocytes of drug-naive schizophrenic patients	2008
Puls, I	Association of reelin with schizophrenia and its effect on the volumetry for schizophrenic patients and healthy controls	2007
Chen, Y	Induction of the reelin promoter by retinoic acid is mediated by Sp1	2007
Siegmund, KD	DNA Methylation in the Human Cerebral Cortex Is Dynamically Regulated throughout the Life Span and Involves Differentiated Neurons	2007
Koros, E	The role of glycogen synthase kinase-3 beta in schizophrenia	2007
Huang, HS	GAD1 mRNA Expression and DNA Methylation in Prefrontal Cortex of Subjects with Schizophrenia	2007
Matsuzaki, H	Disruption of reelin signaling attenuates methamphetamine-induced hyperlocomotion	2007
Liu, LS	Hippocampal CA1 pyramidal cell size is reduced in bipolar disorder	2007

Ruzicka, WB	Selective epigenetic alteration of layer I GABAergic neurons isolated from prefrontal cortex of schizophrenia patients using laser-assisted microdissection	2007
Dong, E	Histone hyperacetylation induces demethylation of reelin and 67-kDa glutamic acid decarboxylase promoters	2007
Kundakovic, M	DNA methyltransferase inhibitors coordinately induce expression of the human reelin and glutamic acid decarboxylase 67 genes	2007
Barr, AM	The reelin receptors VLDLR and ApoER2 regulate sensorimotor gating in mice	2007
Veldic, M	Epigenetic mechanisms expressed in basal ganglia GABAergic neurons differentiate schizophrenia from bipolar disorder	2007
Badea, A	Neuroanatomical phenotypes in the Reeler mouse	2007
Ognibene, E	Neurobehavioural disorders in the infant reeler mouse model: Interaction of genetic vulnerability and consequences of maternal separation	2007
Levenson JM	DNA (cytosine-5) methyltransferase inhibitors: a potential therapeutic agent for schizophrenia.	2007
Tremolizzo L	Epigenetic model in the pharmacologic modulation of vulnerability to schizophrenia	2007
Ognibene, E	Impulsivity-anxiety-related behavior and profiles of morphine-induced analgesia in heterozygous reeler mice	2007
Guidotti, A	S-adenosyl methionine and DNA methyltransferase-I mRNA overexpression in psychosis	2007
Chang, BS	The role of RELN in lissencephaly and neuropsychiatric disease	2007
Gawlowska, M	Disorders of myelination and neuronal migration in the pathogenesis of schizophrenia - looking for new candidate genes	2007
Sharma, RP	Valproic acid and chromatin remodeling in schizophrenia and bipolar disorder: Preliminary results from a clinical population	2006
Krueger, DD	Assessment of cognitive function in the heterozygous reeler mouse	2006
Persico, AM	Polymorphic GGC repeat differentially regulates human reelin gene expression levels	2006
Fatemi, SH	Chronic olanzapine treatment causes differential expression of genes in frontal cortex of rats as revealed by DNA microarray technique	2006
Brigman, JL	Executive functions in the heterozygous reeler mouse model of schizophrenia	2006
Laviola, G	Paradoxical effects of prenatal acetylcholinesterase blockade on neuro-behavioral development and drug-induced stereotypies in reeler mutant mice	2006
Hoareau, C	Postnatal effect of embryonic neurogenesis disturbance on reelin level in organotypic cultures of rat hippocampus	2006
Meyer, U	The time of prenatal immune challenge determines the specificity of inflammation-mediated brain and behavioral pathology	2006
Qui, SF	Cognitive disruption and altered hippocampus synaptic function in Reelin haploinsufficient mice	2006
Botella-Lopez, A	Reelin expression and glycosylation patterns are altered in Alzheimer's disease	2006
Eastwood, SL	Cellular basis of reduced cortical reelin expression in schizophrenia	2006
Simonini, MV	The benzamide MS-275 is a potent, long-lasting brain region-selective inhibitor of histone deacetylases	2006
Costa, E	Epigenetic Targets in GABAergic Neurons to Treat Schizophrenia	2006
Yee, BK	A schizophrenia-related sensorimotor deficit links alpha 3-containing GABA(A) receptors to a dopamine hyperfunction	2005
Dong, E	Reelin and glutamic acid decarboxylase(67) promoter remodeling in an epigenetic methionine-induced mouse model of schizophrenia	2005

Fatemi, SH	Prenatal viral infection in mouse causes differential expression of genes in brains of mouse progeny: A potential animal model for schizophrenia and autism	2005
Grayson, DR	Reelin promoter hypermethylation in schizophrenia	2005
Liu, WS	Oxytocin receptors in brain cortical regions are reduced in haploinsufficient (+/-)reeler mice	2005
Abdolmaleky, HM	Hypermethylation of the reelin (RELN) promoter in the brain of schizophrenic patients: A preliminary report	2005
Fatemi, SH	Reelin signaling is impaired in autism	2005
Mitchell, CP	Histone deacetylase inhibitors decrease reelin promoter methylation in vitro	2005
Tremolizzo, L	Valproate corrects the schizophrenia-like epigenetic behavioral modifications induced by methionine in mice	2005
Roberts, RC	Ultrastructural localization of reelin in the cortex in post-mortem human brain	2005
Veldic, M	In psychosis, cortical interneurons overexpress DNA-methyltransferase 1	2005
Torrey, EF	Neurochemical markers for schizophrenia, bipolar disorder, and major depression in postmortem brains	2005
Noh, JS	DNA methyltransferase 1 regulates reelin mRNA expression in mouse primary cortical cultures	2005
Sharma, RR	Schizophrenia, epigenetics and ligand-activated nuclear receptors: a framework for chromatin therapeutics	2005
Fatemi, SH	GABAergic dysfunction in schizophrenia and mood disorders as reflected by decreased levels of glutamic acid decarboxylase 65 and 67 kDa and Reelin proteins in cerebellum	2005
Kemner, C	In search of neurophysiological markers of pervasive developmental disorders: smooth pursuit eye movements?	2004
Falkai, P	Etiopathogenetic mechanisms in long-term course of schizophrenia	2004
Erbel-Sieler, C	Behavioral and regulatory abnormalities in mice deficient in the NPAS1 and NPAS3 transcription factors	2004
Brown, AS	Serologic-evidence of prenatal influenza in the etiology of schizophrenia	2004
Costa, E	Reelin in brain development and in schizophrenia	2004
Knable, MB	Molecular abnormalities of the hippocampus in severe psychiatric illness: postmortem findings from the Stanley Neuropathology Consortium	2004
Carboni, G	Enhanced dizocilpine efficacy in heterozygous reeler mice relates to GABA turnover downregulation	2004
Perez-Costas, E	Reelin immunoreactivity in the adult sea lamprey brain	2004
Ignatova, N	Characterization of the various forms of the Reelin protein in the cerebrospinal fluid of normal subjects and in neurological diseases	2004
Janusonis, S	Early serotonergic projections to Cajal-Retzius cells: Relevance for cortical development	2004
Veldic, M	DNA-methyltransferase 1 mRNA is selectively overexpressed in telencephalic GABAergic interneurons of schizophrenia brains	2004
Covault, J	Nogo 3 '-untranslated region CAA insertion: failure to replicate association with schizophrenia and demonstration of marked population difference in frequency of the insertion	2004
Manto, MU	Nitric oxide in the cerebellum of mutant mice	2004
Numachi, Y	Psychostimulant alters expression of DNA methyltransferase mRNA in the rat brain	2004
Pappas, GD	Immunocytochemical localization of reelin in the olfactory bulb of the heterozygous reeler mouse: An animal model for schizophrenia	2003
Kovalenko, S	Regio entorhinalis in schizophrenia: More evidence for migrational disturbances and suggestions for a new biological hypothesis	2003

Riedel, A	Reelin-immunoreactive Cajal-Retzius cells: the entorhinal cortex in normal aging and Alzheimer's disease	2003
Scherk, H	The importance of interneurons in schizophrenic and affective disorders	2003
Salinger WL	Behavioral phenotype of the reeler mutant mouse: effects of RELN gene dosage and social isolation.	2003
Larson, J	Olfactory discrimination learning deficit in heterozygous reeler mice	2003
Abraham, H	Reelin-expressing neurons in the postnatal and adult human hippocampal formation	2003
Eastwood, SL	The axonal chemorepellant semaphorin 3A is increased in the cerebellum in schizophrenia and may contribute to its synaptic pathology	2003
Eastwood, SL	Interstitial white matter neurons express less reelin and are abnormally distributed in schizophrenia: towards an integration of molecular and morphologic aspects of the neurodevelopmental hypothesis	2003
Kircher, T	Language, brain and schizophrenia	2003
Stone, J	Monitoring treatment effects	2003
Tremolizzo, L	An epigenetic mouse model for molecular and behavioral neuropathologies related to schizophrenia vulnerability	2002
Fatemi, SH	Glutamic acid decarboxylase 65 and 67 kDa proteins are reduced in autistic parietal and cerebellar cortices	2002
Rodriguez, MA	In Patas monkey, glutamic acid decarboxylase-67 and reelin mRNA coexpression varies in a manner dependent on layers and cortical areas	2002
Chen, Y	On the epigenetic regulation of the human reelin promoter	2002
Fatemi, SH	Reduced blood levels of reelin as a vulnerability factor in pathophysiology of autistic disorder	2002
Ballmaier, M	Preferential alterations in the mesolimbic dopamine pathway of heterozygous reeler mice: an emerging animal-based model of schizophrenia	2002
Martinez-Cerdeno, V	Reelin immunoreactivity in the adult neocortex: A comparative study in rodents, carnivores, and non-human primates	2002
Fatemi, SH	Prenatal viral infection leads to pyramidal cell atrophy and macrocephaly in adulthood: Implications for genesis of autism and schizophrenia	2002
Costa, E	The heterozygote reeler mouse as a model for the development of a new generation of antipsychotics	2002
Fatemi, S	The role of Reelin in pathology of autism	2002
Zhang, H	Reelin gene alleles and susceptibility to autism spectrum disorders	2002
Fatemi, SH	Dysregulation of Reelin and Bcl-2 proteins in autistic cerebellum	2001
Fatemi, SH	Altered levels of Reelin and its isoforms in schizophrenia and mood disorders	2001
Chen, CH	Identification of a molecular variant at the promoter region of human reelin gene and association study with schizophrenia.	2001
Pappas, GD	Reelin in the extracellular matrix and dendritic spines of the cortex and hippocampus: a comparison between wild type and heterozygous reeler mice by immunoelectron microscopy	2001
Pesold, C	A reelin deficit may underlie the neuroanatomical abnormalities observed in the brains of schizophrenic patients	2001
Knable MB	Multivariate analysis of prefrontal cortical data from the Stanley Foundation Neuropathology Consortium.	2001
Petek, E	Disruption of a novel gene (IMMP2L) by a breakpoint in 7q31 associated with Tourette syndrome	2001
Liu, WS	Down-regulation of dendritic spine and glutamic acid decarboxylase 67 expressions in the reelin haploinsufficient heterozygous reeler mouse	2001
Fatemi, SH	Reelin mutations in mouse and man: from reeler mouse to schizophrenia, mood disorders, autism and lissencephaly	2001

Guidotti, A	Decrease in reelin and glutamic acid decarboxylase(67) (GAD(67)) expression in schizophrenia and bipolar disorder - A postmortem brain study	2000
Fatemi, SH	Reduction in Reelin immunoreactivity in hippocampus of subjects with schizophrenia, bipolar disorder and major depression	2000
Rodriguez, MA	Colocalization of integrin receptors and reelin in dendritic spine postsynaptic densities of adult nonhuman primate cortex	2000
Grayson, DR	Reelin (RELN) gene RFLP analysis in pedigrees of schizophrenia and bipolar patients with psychosis.	1999
Tueting, P	The phenotypic characteristics of heterozygous reeler mouse	1999
Fatemi, SH	Defective corticogenesis and reduction in Reelin immunoreactivity in cortex and hippocampus of prenatally infected neonatal mice	1999
Impagnatiello, F	A decrease of reelin expression as a putative vulnerability factor in schizophrenia	1998
Impagnatiello, F	Reelin mRNA is significantly decreased (similar to 50%) in several areas of schizophrenic brain	1998

Table S3a Genetic model studies

Animal model	Author	Specie	Sex	Age	Behavior characterization	Methods	Analysis	Results	Rescue experiments	Reelin alteration
HRM	Nullmeier et al. 2011	Mouse	Male and female	28-week-old		HRM mouse	Immunocitochemistry (GAD67, PV, TH, 5-HT-T). Cell count and area measurement.	Decreased of GAD67-positive cells in HP and PV-positive cells in CA1 and CA2. Impairment of hippocampal GABAergic functioning.		50% reelin expression
	Texeira et al. 2011	Mouse	Male and female	Not specified	Open field, novelty suppressed feeding, forced swim test and PPI.	HRM mouse	Behavioral characterization with or without corticosterone, cocaine sensitization and PPI with NMDA antagonist treatment.	No differences between groups in different analysis.		50% reelin expression
	van den Buuse et al. 2012	Mouse	Male and female	10-12-week-old	Locomotor hyperactivity, PPI	HRM mouse	Western blot (NMDAr subunits). Behavior characterizacion.	Increased MK-801-induced locomotor hyperactivity in males. Altered startle reflex caused by effect of MK-801. Upregulation of NR1 subunits and down-regulation of NR2C subunits in frontal cortex.		50% reelin expression
	Rogers et al. 2013	Mouse	Male and female	Not specified	Fear conditioning and PPI	HRM mouse injected with reelin	Golgi staining and morphology studies. Immunohistochemistry (GAD67). Brain slices electrophysiology. Behavioral characterization.	Reduced dendritic spine density and synaptic plasticity. Impaired behavior (associative learning and memory, PPI) in HRM. Increased GAD67 expression and altered dendritic spine morphology in HRM injected with Reelin.	Reelin injection	50% reelin expression
	Nullmeier et al. 2014	Mouse	Male	24-28-week-old		HRM mouse	Tyrosin hydroxylase (TH)-immunoreactive and serotonin (5-HT) fibers in PFC, HP and striatum. Immunohistochemistry.	Increased tyrosine hydroxylase immunoreactive densities in HP and decreased in nucleus accumbens.		50% reelin expression
	Magliaro et al. 2016	Mouse	Male and female	2-month-old		HRM mouse	Number of Purkinje neurons and their topology in the cerebellar vermis	Reduced Purkinje neurons density in male and female HRM mice. Larger Purkinje neurons diameter in male HRM than female. Chaotic organization of Purkinje neurons inHRM.		50% reelin expression

Loss of reelin function	Sakai et al. 2016	Mouse	Male	11-week-old	Open field, anxiety test, three-chamber, t-maze, Barnes maze, PPI and fear conditioning.	C-terminal reelin knock in mouse	Immunohistochemistry and western blot.<br>Behavioral characterization.<br>Body weight control.	Hyperactivity and reduced anxiety and social behavior.<br>Impairment of working memory.<br>Attenuated Reelin signaling in cortex and HP.<br>Decreased body weight.		↓ reelin signaling in cortex and HP
	Pahle et al. 2020	Mouse	Male	15-week-old	Elevated plus maze, open field, Morris water maze.	Reelin knock out mouse (inhibitory interneurons)	Immunohistochemistry (NeuN, KI67, DCX, BrdU, GFAP, Nestin, GAD67, PV, RFP, CB1, Sox2, CCK, GFP and Calbindin).<br>ISH (Ndnf, Rgs8, Rorb and Etv1).<br>Western blot (reelin).<br>Behavioral characterization.	Decreased reelin expression in neocortex and dentate gyrus.<br>In dentate gyrus, increased Cajal Retzius cells number that express reelin, increased CB1 expression and decreased GFAP-positives astrocytes.<br>NO DICE NADA DE COMPORTAMIENTO.		↓ reelin expression in neocortex and dentate gyrus
ADAMTS KO	Ogino et al. 2017	Mouse	Not specified	E18.5		ADAMTS3 knock out mouse	ISH and immunohistochemistry (ADAMTS3 and reelin). Golgi staining. Western blot.	Recombinant ADAMTS3 cleaves Reelin at the N-t site.<br>Decreased N-t cleavage of Reelin in ADAMTS3 KO mice.<br>Increased dendritic branching and elongation in cortex of conditional KO mice.		↓ reelin cleavage
	Yamakage et al. 2019	Mouse	Male and female	P60		ADAMTS2 knock out mouse	qRT-PCR (ADAMTS2 and ADAMTS3).<br>ISH (ADAMTS2).	Recombinant ADAMTS2 cleaves Reelin at the N-t site.<br>The disintegrin domain is necessary for the Reelin-cleaving activity. ADAMTS2 is necessary for the N-t cleavage and inactivation of Reelin.		↓ reelin cleavage and reelin levels in HP
Vldlr overexpression	Iwata et al. 2012	Rat	Not specified	2-3-month-old	Open field, locomotor activity, radial maze, social interaction and elevated plus maze.	Overexpression of Vldlr in transgenic rat	RT-qPCR and western blot (Vldlr).<br>Histology (NeuN).<br>Behavioral characterization.	Increased locomotor activity.<br>Impairment of spatial working memory.<br>Vldlr levels involved in locomotor activity and memory functions.		Altered reelin signaling (decreased Dab1 levels in PFC and cerebellum)
Reelin antisense	Brosda et al. 2011	Rat	Male	P43, p93	PPI, startle reflex, working memory, novel object recognition, locomotor activity.	Reelin antisense (knock down) in PFC of rats	Western blot (reelin in mPFC).<br>Behavioral characterization.	Impairment of PPI, spatial memory and novel object recognition. Selective alteration of mPFC.<br>Low reelin protein expression		↓ reelin expression

Table S3b Environmental model studies

Animal model	Author	Specie	Sex	Age	Behavior characterization	Methods	Analysis	Results	Rescue experiments	Reelin alteration
PCP	Radonjić et al. 2013	Rat	Male	P70		Rat injected with 10mg/kg at P2, P6, P9 and P12.	Immunohistochemical staining (NeuN, PV, calretinin, somatostatin, reelin, VGAT). Western blot (NRG-1).	Reduced neurons density in CA3 and dentate gyrus and interneuronal populations. Decreased reelin- and somatostatin- positive cells PFC and HP. PCP increased NRG-1 protein expression in cortex and HP.		↓ reelin positives cells in PFC and HP
MAM	Matricon et al. 2010	Rat	Male and female	6-month- old		Rat exposed at E17.	Immunohistochemistry (NeuN, GFAP, reelin). Morphometric analysis. Methylation analysis. RNA expression.	Reduced entorhinal cortex volume, HP and mediodorsal thalamus (decreased neuronal soma). Laminar disorganization and neuronal clusters in entorhinal cortex. Reduced reelin methylation in HP.		↓ reelin methylation in HP
Influenza	Fatemi et al. 2017	Mouse	Male	P0, P14, P35, P56		Mouse offspring. Maternal exposure to H1N1 at E16.	Western blot (reelin, GABAr, FMRP, mGluR5, GAD65/67, Vldlr) in cerebellum.	Altered FMRP expression (increased at P0 and P14), VLDLR (decreased at P14, increased at P35) and GAD65/67 (increased at P35). Impaired FMRP, glutamatergic and reelin signaling.		Impaired reelin signaling
Poly(I:C)	Harvey & Boksa 2012	Mouse	Male and female	P28		Mouse offspring. Maternal injection of 20 mg/kg at GD9.	Immunohistochemistry (NeuN, reelin and GAD67). Body weight control.	Decreased number of reelin- positive cells in dorsal stratum oriens. Increased GAD67 expression in ventral stratum oriens in females. Weight gain differences.		↓ reelin positives cells in stratum oriens
	Ratnayake et al. 2012	Mouse	Male and female	P1, P100	Open field, novel object recognition, elevated plus maze, rotarod.	Mouse offpsring. Maternal injection of 0,5mg/kg at GD20.	Immunohistochemistry and histology (reelin, GFAP, Iba1). Behavioral characterization.	Impairment of non-spatial memory, learning and motor activity. Reduced reelin-positive cells and increased GFAP expression, and increased number of activated microglia in HP.		↓ reelin positives cells in HP

	Giovanoli et al. 2014	Mouse	Male	P70		Mouse offpsring. Maternal injection of 1 mg/kg at GD9 with sub-cronic stress.	Immunohistochemistry (PV and reelin).	Decreased number of GABAergic interneurons in ventral dentate gyrus (exposure to Poly(I:C) and stress).<br>Stress increases reelin expression in control offspring and decreases reelin expression in the rodent model (exposure to Poly(I:C) and stress).		↓ reelin positives cells in HP
LPS	Ghiani et al. 2011	Rat	Male and female	E18		Rat offspring (E18). Maternal injection of 200ug at GD15 and GD16.	Immunohistochemistry and staining.	Increased thickness of the cortical plate.<br>Abnormal distribution of immature neuronal markers and lower expression of progenitor markers.<br>Reduced levels of Reelin and GLAST.<br>Alteration of neuronal maturation. Increased. GFAP levels by 40% after 72h of injection.		↓ reelin expression
	Nouel et al. 2012	Rat	Male	P14, P28		Rat injected with 100ug/kg at E15 and E16.	Cell count of GAD67-andreelin- immunoreactive neurons in HP.<br>Western blot (reelin and GAD67).	Reduced GAD67-positive cells in dentate gyrus and CA1.<br>Decreased reelin-positives cells in dentate gyrus and CA1.		↓ reelin positives cells in HP
Maternal deprivation	Aksic et al. 2021	Rat	Male and female	P60		Rat offsprings with maternal deprivation from P9 to P10.	Immunohistochemistry (PV and reelin).<br>Dell death assay.	Reduced number of PV positive interneurons in CA1 (HP) and PFC. Reduced number of reelin positive interneurons in CA1 and CA3 (HP), but unaltered in neocortex.<br>No differences in cell death.		↓ reelin positives cells in HP
PRS	Matrisciano et al. 2013	Mouse	Male	P1, p7, p14, p60	Open field, social behavior, PPI and fear conditioning	Mouse offspring exposed to PRS (30 minutes, 2 times per day, from E7 to E21)	RT-PCR (DNMT1 and DNMT3a of PFC and HP).<br>Western blot (DNMT1, reelin and GAD67).<br>Behavioral characterization.	Increased mRNA levels of DNMT1 and DNMT3a (P1 to p60) in the frontal cortex and HP.<br>DNMT overexpression was associated with a decreased in reelin protein expression in frontal cortex.<br>Hyperactivity and impairment of social interaction, prepulse inhibition and fear conditioning, corrected with the administration of valproic acid or clozapine.	Clozapine, Valproic acid	↓ reelin expression in frontal cortex. ↑ reelin promoter methylation
	Palacios-García et al. 2015	Rat	Male and female	E20, P60	Open field, elevated plus maze, passive avoidance, Morris water maze (P60)	Rat offspring exposed to PRS (2 hours, 1 time per day).	Immunohistochemistry (reelin and NeuN).<br>Cell count of reelin- and NeuN-immunoreactive neurons. Western blot (reelin, Dab1, CDK5, PHF-1 and tubuline).<br>Behavior characterization.	Decreased density of reelin- positive cells in cortex.<br>Decreased reelin protein and gene expression.<br>79% decrease in reelin immunoreactivity in prefrontal cortex. Increased DNA methylation levels of Reelin promoter.<br>Increased Dab1 adapter protein and decreased phosphorylation of H1 histone.<br>Excessive spontaneous locomotor activity, high anxiety levels and impaired learning and memory consolidation.		↓ reelin positives cells in cortex. ↓ reelin expression. ↑ reelin promoter methylation

	Dong et al. 2016	Mouse	Male	P75	Open field, three-chamber (P75)	Mouse offspring exposed to PRS (45 minutes, 3 times per day, from E7 to E21) treated with clozapine (5mg/kg, twice a day for 5 days).	Behavior characterization. RT-qPCR (Nse, NeuN). Western blot (DNMT1). Methylated DNA immunoprecipitation.	Clozapine and valproic acid, but not haloperidol, correct the behavioral impairments in PRS mice. Clozapine and valproic acid, but not haloperidol, hypermethylation of psychiatric disorder-related genes. Clozapine treatment corrected the elevated DNMT1 protein expression level and reduced the DNMT1 binding to reelin promoter.	Clozapine, Valproic acid	↓ reelin expression in frontal cortex. ↑ reelin promoter methylation
Reelin injection	Ishii et al. 2015a	Mouse	Male	6-7-week-old	PPI, novel object recognition.	Reelin injection in lateral ventricle of mouse and PCP (1mg/kg) 30 min before behavior test	Immunohistochemistry (VLDLR, CaMKII and PV). Behavioral characterization.	Reelin injection prevents PCP- induced behavioral phenotyes, increases dendritic spines density and synaptic plasticity, and improves memory and spatial learning.	Reelin injection	Reelin injection
	Ishii et al. 2015b	Mouse	Male	P14.5, 7-month-old	Y- maze, three chamber test (P14.5)	Mice with focal heterotopias in somatosensory cortex	In situ hybridation. Behavioral characterization.	Impaired spatial working memory and low competitive dominant behavior. Decreased immediate early gene expression.		No differences
	Sawahata et al. 2021	Mouse	Male	7-9-week-old	NOR, PPI, y-maze.	Reelin injection in mPFC of MK-801 model (0,15mg/kg 30 min before behavior test).	Immunohistochemistry (c-Fos positive cells in mPFC). Behavioral characterization.	Reelin injection prevents MK- 801-induced impairment of recognition memory. No effect of treatment in sensory-motor gating or short term memory deffects. Reelin treatment reduced number of c-Fos positive cells to control levels in mPFC.	Reelin injection	Reelin injection
High fat diet	Roberts et al. 2019	Mouse	Male	12-24-week-old		Mouse with high fat diet (12-16 weeks)	qPCR and western blot (ApoER2 and VLDLR). ISH (RELN, VLDLR and ApoER2).	Reduced expression of ApoER2 and VLDLR and increased levels of reelin protein in hypothalamus.		↑ reelin expression

Table S3c Gene x environment interaction model studies

Animal model	Author	Specie	Sex	Age	Behavior characterization	Methods	Analysis	Results	Rescue experiments	Reelin alteration
Social isolation	Ko et al. 2016	Rat	Male and female	3 nd 7-week-old	Locomotion and PPI	Rat offspring isolated at P21 and mated with social rats. Offsprings of 2 next generation evaluated.	qPCR (monoamines and schizophrenia-related genes in mPFC and HP). Behavioral characterization.	Impaired PPI in second generation of rats. Lower levels of dopamine and serotonine in mPFC and HP in the third generation. Female isolation rats presented elevated reelin gene expression levels in PFC with respect to non isolation rats in the third generation.		↑ reelin gene expression levels in PFC
HRM	Romano et al. 2013	Mouse	Male	P35	Locomotion (P35)	HRM mouse injected with nicotine (1mg/kg for 6 days)	RT-PCR (reelin, GAD67 and BDNF). Behavior characterizacion and its reponses to nicotine.	Decreased reelin and GAD67 gene expression in PFC, HP, cerebellum and striatum. Hyperactivity in HRM. Reversion of phenotypes in HRM treated with nicotine. Increased BDNF gene expression of HRM and HRM treated with nicotine.	Nicotine	50% reelin expression
	Hill et al. 2013	Mouse	Male and female	Not specified		HRM mouse	Western blot (BDNF, TrkB, fosforilated TrkB and MAPK).	Increased BDNF levels in female HP and decreased phosphorylated ERK1 levels. Ovariectomy decreases BDNF expression in HP.		50% reelin expression
	Howell & Pillai 2014	Mouse	Not specified	E18, 1 month-old, 3-month-old	PPI, Y-maze, open field PFC and HP volume.	HRM mouse with prenatal hypoxia (9% oxigen, 2 hours at E17)	Levels of HIF-1a, VEGF, VEGFR2/Flk1 and GR analysis. Behavioral characterization.	Anxiety-like behavior in HRM and wt with hypoxia. Increased protein levels of VEGF in HP in HRM and wt with hypoxia. Increased protein levels of GR in Frontal Cortex in HRM and wt with hypoxia. Lower corticosterone serum levels in HRM and wt with hypoxia. Increased levels of HIF-1a and VEGF in the forebrain in HRM with hypoxia (E18). Increased GR levels in the forebrain in HRM with hypoxia (E18, 3-month-old).		50% reelin expression
	Romano et al. 2014	Mouse	Male	P37-42	Light/dark test, openfield, hole-board test, T-maze.	HRM mouse with oral nicotine stimulation (10mg/l, from P37 to P42)	Behavior characterization. RT-PCR (Reelin, GAD67).	Nicotine restores impaired behavior (exploratory behavior and poor cognitive performance). Reelin and GAD67 mRNA expression to WT levels in PFC, HP, cerebellum and striatum.	Nicotine	50% reelin expression

	Schroeder et al. 2015	Mouse	Male and female	11-week-old	Y- maze, novel object recognition, social test and PPI	HRM mouse exposed to stress (corticosterone treatment)	Reelin expression in PFC and HP.<br>Behavioral characterization.	Increased reelin expression in PFC of female HRM treated with corticosterone..<br>Impaired spatial memory in HRM with corticosterone.<br>Altered PPI in male WT exposed to corticosterone, but not in male HRM.		50% reelin expression
	Mullen et al. 2016	Mouse	Male	P90		HRM mouse offspring. Prenatal infection with 20mg/kg of CPO (GD13.5)	Western blot (reelin).<br>Golgi staining.<br>Nissl staining.<br>DAPI staining.	Decreased full lenght and cleaved Reelin protein an altered cellular complexity and dendritic spine organization in CPO exposed mice.<br>Reduced reelin expression by prenatal pesticide exposure can alter the shape and connectivity of neurons in several brain regions.		50% reelin expression
	Howell & Pillai 2016	Mouse	Not specified	6-month-old	PPI, open field and y-maze.	HRM mouse with prenatal hypoxia (9% oxigen, 2 hours at E17)	Western blot (reelin, VEGF, Flk1 and GR).<br>MRI imaging.<br>Behavioral characterization.	Increased frontal cortex volume in prenatal hypoxia mice.<br>Decreased frontal cortex volume in HRM with prenatal hypoxia.<br>Decreased reelin expression in frontal cortex in prenatal hypoxic miceand HRM, and HP in HRM.<br>Decreased HIF- 1a levels in frontal cortex anddecreased serum VEGF in HRM.		50% reelin expression
	Notaras et al. 2017	Mouse	Male and female	11-week-old	Methanphetamine-induced locomotor hyperactivity, PPI, y-maze and forced swim test.	HRM mouse with oral corticosterone (50mg/l)	Behavioral characterization.	Decreased PPI in HRM. Increased immobility in forced swim test and decreased novel arm preference in y-maze in HRM with corticosterone treatment.		50% reelin expression

Abbreviations: phencyclidine (PCP), methylazoxymethanol (MAM), polyinosinic:polycytidylic acid (Poly(I:C)), lipopolysaccharide (LPS), prenatal restraint stress (PRS), heterozygous reeler mouse (HRM), overexpression (OE).

Quality assessment procedures S4

Year	Author	Title	Was the allocation sequence adequately generated and applied?	Were the groups similar at baseline or were they adjusted for confounders in the anylisis?	Was the allocation adequately concealed?	Were the animals randomly housed during the experiment?	Were the caregivers and/or investigators blinded from knowledge which intervention each animal received during the experiment?	Were animals selected at random for outcomes assessment?	Was the outcome assessor blinded?	Were incomplete outcome data adequately addressed?
			Risk of bias	Risk of bias	Risk of bias	Risk of bias	Risk of bias	Risk of bias	Risk of bias	Risk of bias
2010	Matricon	Neuropathological and Reelin Deficiencies in the Hippocampal Formation of Rats Exposed to MAM; Differences and Similarities with Schizophrenia	Unclear	Unclear	Unclear	Low	Low	Unclear	Low	Unclear
2011	Texeira	Overexpression of Reelin prevents the manifestation of behavioral phenotypes related to schizophrenia and bipolar disorder	Low	Low	Low	Low	Low	Low	Low	Low

2011	Brosda	Impairment of cognitive performance after reelin knockdown in the medial prefrontal cortex of pubertal or adult rats	Unclear	Low	Unclear	Low	Unclear	Low	Low	Unclear
2011	Ghiani	Early effects of lipopolysaccharide induced inflammation on foetal brain development in rat	Unclear	Unclear	Unclear	Low	Low	Low	Low	Unclear
2011	Nouel	Prenatal exposure to bacterial endotoxin reduces the number of GAD67 and reelin immunoreactive neurons in the hippocampus of rat offspring	Low	Unclear	Low	Low	Unclear	Low	Low	Unclear
2011	Nullmeier	Region specific alteration of GABAergic markers in the brain of heterozygous reeler mice	Unclear	Unclear	Unclear	Low	Unclear	Unclear	Low	Low

2012	Ratnayake	Behavior and hippocampus specific changes in spiny mouse neonates after treatment of the mother with the vial mimetic PolyIC at mid-pregnancy	Low	Low	Low	Low	Low	Unclear	Low	Unclear
2012	Harvey	A stereological comparison of GAD67 and reelin expression in the hippocampal striatum oriens of offspring from two mouse models of maternal inflammation during pregnancy	Unclear	Unclear	Low	Low	Low	Unclear	Low	Low
2012	Iwata	Vldr overexpression causes hyperactivity in rats	Unclear	Unclear	Unclear	Low	Low	Unclear	Low	Low
2012	Matrisciano	Epigenetic modifications of GABAergic interneurons are associated with the schizophrenia-like phenotype induced by prenatal stress in mice	Low	Unclear	Unclear	Low	Low	Unclear	Low	Low

2012	Van der buuse	Altered N-methyl-d-aspartate receptor function in reelin heterozygous mice: Male-female differences and comparison with dopaminergic activity	Low	Unclear	Unclear	Low	Unclear	Unclear	Low	Low
2013	Hill	Sex-dependent alterations in BDNF TrkB signaling in the hippocampus of reelin heterozygous mice: a role for sex steroid homones	Unclear	Unclear	Unclear	Low	Unclear	Unclear	Low	Unclear
2013	Radonjic	Perinatal phencyclidine administration decreases the density of cortical interneurons and increases the expression of neuregulin-1	Unclear	Low	Low	Low	Unclear	Low	Unclear	Unclear
2013	Rogers	Reelin supplementation recovers sensorimotor gating, synaptic plasticity and associative learning deficits in the heterozygous reeler mice	Low	Low	Unclear	Unclear	Unclear	Low	Unclear	Unclear

2013	Romano	Nicotine restores wt-like levels of reelin and GAD67 gene expression in brain of heterozygous reeler mice	Low	Unclear	Unclear	Low	Unclear	Unclear	Unclear	Unclear
2014	Ishii	Reelin has a preventive effect on phencyclidine-induced cognitive and sensory-motor gating deficits	Low	Unclear	Unclear	Low	Unclear	Unclear	Unclear	Unclear
2014	Nullmeier	Alterations in the hippocampal and striatal catecholaminergic fiber densities of heterozygous reeler mice	Low	Unclear	Low	Unclear	Unclear	Unclear	Unclear	Unclear
2014	Giovanoli	Single and combined of prenatal immune activation and peripubertal stress on parvalbumin and reelin expression in the hippocampal formation	Low	Unclear	Unclear	Low	Unclear	Unclear	Unclear	Low

2014	Romano	Nicotine exposure during adolescence: cognitive performance and brain gene expression in adult heterozygous reeler mice	Unclear	Unclear	Unclear	Low	Unclear	Low	Unclear	Low
2015	Howell	Long term effects of prenatal hypoxia on schizophrenia-like phenotype in heterozygous reeler mice	Unclear	Unclear	Unclear	Low	Unclear	Low	Low	Low
2015	Schoeder	Gene-environment interaction of reelin and strees in cognitive behaviors in mice: implications for schizophrenia	Low	Low	Unclear	Low	Unclear	Unclear	Low	Low
2015	Palacios García	Prenatal stress down regulates reelin expression by methylation of its promoter and induces adult behavioral impairments in rats	Unclear	Low	Unclear	Low	Unclear	Unclear	Low	Low
2015	Ishii	Neuronal heterotopias affect the activities of distant brain areas and lead to behavioral deficits	Low	Low	Unclear	Low	Low	Unclear	Low	Low

2016	Chih yuan	Sensorimotor gating deficits are inheritable in an isolation-rearing paradigm in rats	Low	Low	Unclear	Unclear	Unclear	Low	Low	Unclear
2016	Dong	Behavioral and molecular neuroepigenetic alterations in prenatally stressed mice: relevance for the study of chromatin remodeling properties of antipsychotic drugs	Low	Low	Low	Low	Unclear	Low	Low	Unclear
2016	Fatemi	The effects of prenatal H1N1 infection at E16 on FMRP, glutamate, GABA and reelin signaling systems in developing murine cerebellum	Low	Low	Unclear	Low	Unclear	Low	Unclear	Unclear
2016	Magliaro	The number of Purkinje neurons and their topology in the cerebellar vermis of normal and reln haplodeficient mouse	Unckear	Low	Unclear	Low	Unclear	Low	Unclear	Low

2016	Müllen	A complex interaction between reduced reelin expression and prenatal organophosphate exposure alters neuronal cell morphology	Unclear	Unclear	Unclear	Low	Unclear	Unclear	Low	Low
2016	Sakai	Mice that lack the C terminal region of reelin exhibit behavioral abnormalities related to neuropsychiatric disorders	Low	Low	Unclear	Low	Unclear	Unclear	Low	Low
2017	Notaras	Interaction of reelin and stress on immobility in the forced swim test but not dopamine-mediated locomotor hyperactivity or prepulse inhibition disruption: relevance to psychotic and mood disorders	Low	Unclear	Low	Low	Unclear	Low	Unclear	Low
2017	Ogino	Secreted metalloproteinase ADAMTS3 inactivates reelin	Low	Unclear	Unclear	Low	Unclear	Low	Low	Low

2019	Roberts	Reelin is modulated by diet induced obesity and has direct actions on arcuate proopiomelanocortin neurons	Unclear	Low	Low	Low	Low	Low	Low	Low
2019	Yamakage	A disintegrin and metalloproteinase with thrombospondin motifs 2 cleaves and inactivates reelin in the postnatal cerebral cortex and hippocampus, but not in the cerebellum	Low	Low	Unclear	Low	Low	Unclear	Low	Low
2020	Pahle	Selective inactivation of reelin in inhibitory interneurons leads to subtle changes in the dentate gyrus but leaves cortical layering and behavior unaffected	Low	Unclear	Low	Low	Low	Low	Low	Low
2021	Sawahata	Microinjection of Reelin into the mPFC prevents MK-801-induced	Low	Low	Unclear	Low	Unclear	Low	Low	Low

2021	Aksic	Maternal deprivation in rats decreases the expression of interneuron markers in the neocortex and hippocampus	Low	Low	Unclear	Low	Unclear	Low	Low	Low
